# ALVAC-HIV B/C candidate HIV vaccine efficacy dependent on neutralization profile of challenge virus and adjuvant dose and type

**DOI:** 10.1371/journal.ppat.1008121

**Published:** 2019-12-03

**Authors:** Luca Schifanella, Susan W. Barnett, Massimiliano Bissa, Veronica Galli, Melvin N. Doster, Monica Vaccari, Georgia D. Tomaras, Xiaoying Shen, Sanjay Phogat, Ranajit Pal, David C. Montefiori, Celia C. LaBranche, Mangala Rao, Hung V. Trinh, Robyn Washington-Parks, Namal P. M. Liyanage, Dallas R. Brown, Frank Liang, Karin Loré, David J. Venzon, William Magnanelli, Michelle Metrinko, Josh Kramer, Matthew Breed, Galit Alter, Ruth M. Ruprecht, Genoveffa Franchini

**Affiliations:** 1 Animal Models and Retroviral Vaccines Section, Vaccine Branch, Center for Cancer Research, National Cancer Institute, Bethesda, Maryland, United States of America; 2 Novartis Vaccines and Diagnostics, Inc, Cambridge, Massachusetts, United States of America; 3 Duke Human Vaccine Institute, Duke University, Durham, North Carolina, United States of America; 4 Sanofi Pasteur, Swiftwater, Pennsylvania, United States of America; 5 Advanced BioScience Laboratories, Inc., Rockville, Maryland, United States of America; 6 U.S. Military HIV Research Program, Walter Reed Army Institute of Research, Silver Spring, Maryland, United States of America; 7 U.S. Military HIV Research Program, Henry M. Jackson Foundation for the Advancement of Military Medicine, Bethesda, Maryland, United States of America; 8 Karolinska Institute, Stockholm, Sweden; 9 Biostatistics and Data Management Section, National Cancer Institute, National Institutes of Health, Bethesda, Maryland, United States of America; 10 Laboratory Animal Sciences Program, Frederick National Laboratory for Cancer Research, Leidos Biomedical Research, Inc., Frederick, Maryland, United States of America; 11 Ragon Institute of MGH, MIT, and Harvard Cambridge, Boston, Massachusetts, United States of America; 12 Texas Biomedical Research Institute, San Antonio, Texas, United States of America; University of Zurich, SWITZERLAND

## Abstract

The ALVAC-HIV clade B/AE and equivalent SIV-based/gp120 + Alum vaccines successfully decreased the risk of virus acquisition in humans and macaques. Here, we tested the efficacy of HIV clade B/C ALVAC/gp120 vaccine candidates + MF59 or different doses of Aluminum hydroxide (Alum) against SHIV-Cs of varying neutralization sensitivity in macaques. Low doses of Alum induced higher mucosal V2-specific IgA that increased the risk of Tier 2 SHIV-C acquisition. High Alum dosage, in contrast, elicited serum IgG to V2 that correlated with a decreased risk of Tier 1 SHIV-C acquisition. MF59 induced negligible mucosal antibodies to V2 and an inflammatory profile with blood C-reactive Protein (CRP) levels correlating with neutralizing antibody titers. MF59 decreased the risk of Tier 1 SHIV-C acquisition. The relationship between vaccine efficacy and the neutralization profile of the challenge virus appear to be linked to the different immunological spaces created by MF59 and Alum via CXCL10 and IL-1β, respectively.

## Author summary

The ALVAC-based clade C /gp120 combination is currently being tested for efficacy and licensures in South Africa in the HVTN702 HIV vaccine trial using the MF59 adjuvant rather than Alum with the intent of inducing higher neutralizing antibodies and T cell responses. Here, we present studies in 71 macaques that evaluated the relative efficacy of identical ALVAC-HIV B/C HIV vaccines with Alum or MF59 following challenge exposure to SHIV-clade C viral stocks that differed in their neutralization profiles. Our results demonstrated that use of the proprietary Novartis Alum at lower doses affects the proportion of mucosal V2-specific IgG and IgA, and that IgAs and IgGs were respectively associated with an increased or decreased risk of virus acquisition. In contrast, animals immunized with the MF59 regimen had no V2 mucosal responses, and a high level of serum neutralizing antibodies (Tier 1) to easy to neutralize viruses that were associated with a decreased risk of Tier 1 SHIV-C acquisition. *In vitro* studies suggest the hypothesis that the low doses of proprietary Novartis Alum used in our studies may be the underlying reason for the decreased vaccine efficacy, via lower inflammasome activation and IL-1β production.

## Introduction

The development of a fully protective vaccine against HIV has proven challenging, and only four (gp120, recombinant Ad5, DNA plus recombinant Ad5, and ALVAC-based regimens) out of more than 250 HIV vaccine candidates have even been tested in phase IIb/III efficacy studies [1–5]. The RV144 clinical trial (NCT00223080) is the only one so far to afford limited vaccine efficacy (31.2%), using a regimen based on a combination of the recombinant canarypox vector ALVAC-HIV vCP1521 expressing clade B Gag-Pro and clade E gp120-TM with the recombinant gp120 B/AE proteins adjuvanted in Alum Alhydrogel [6]. Analysis of RV144 identified binding antibodies to the HIV envelope variable region 2 (V2), CD4^+^ T cells, and antibody-dependent cell-mediated cytotoxicity (ADCC) in individuals with low levels of IgA as inverse correlates of risk of HIV acquisition [7, 8]. Crucially, half of the 35 million documented cases of HIV infection worldwide [9] are caused by HIV-1 clade C (HIV-C) [10], and the phase IIb/III HVTN702 trial is now underway in South Africa to investigate effectiveness of the ALVAC-based vaccine against HIV-C [11].

HVTN702 builds on the vaccine used in RV144 and HVTN100 [12], but the South African study is distinguished by several important elements. Foremost, HVTN702 tests ALVAC gp120/MF59 clade C vaccines in a high-risk population. Indeed, 7.1 million people were reported to be living with HIV-1 in South Africa in 2016, with a prevalence of 18.9% in the general adult population and 270,000 new infections [13, 14]. By contrast, the vaccinees enrolled in RV144 were at low risk for HIV-1 acquisition. In the same year, 450,000 people were living with HIV-1 in Thailand, with a prevalence of 1.1% and 6,400 new infections [15]. The different rate of infection between these populations is jarring, and HIV prevalence and incidence were respectively 17 and 42 times higher in South Africa than in Thailand [16]. Further separating HVTN702 and RV144 are the recombinant ALVAC vCP2438, expressing the 96ZM651 HIV clade C envelope, and the two clade C TV1 and 1086 gp120 protein boosts. Finally, the use of adjuvant differs between these trials, with HVTN702 employing an MF59 oil-in-water emulsion instead of the Alum Alhydrogel used in RV144.

Our prior studies in the SIV_mac251_ macaque model with Alum Alhydrogel and MF59 adjuvants demonstrated that the ALVAC/gp120/Alum Alhydrogel regimen (SIV-Alum^Alh12.5^) recapitulated the limited efficacy of RV144 and identified V2 antibodies and CD4^+^ T cells as inverse correlates of risk against the neutralization-resistant Tier 2 SIV_mac251_, as they were in humans [17, 18]. However, an otherwise identical vaccine regimen using MF59 as adjuvant (SIV-MF59) unexpectedly did not reduce the risk of SIV_mac251_ acquisition, despite its higher immunogenicity [18, 19].

The predictive value of macaque models in assessing the efficacy of HIV vaccine candidates in humans remains uncertain, and further testing is necessary to determine its usefulness. Thus, we investigated the efficacy of the clade B/C ALVAC-HIV gp120 vaccine regimens in macaques with a gp120 protein boost formulated in MF59 or with varying Alum/gp120 ratios of the proprietary Alum^N^ adjuvant (Novartis). We found that the alum adjuvant at a ratio of 1.9:1 gp120 (Alum^N1.9^; Study 1) induced V2-specific IgA that correlated with an increased risk of acquisition of Tier 2 SHIV-C in vaccinated animals. The Alum^N1.9^ regimen did not protect from Tier 2 SHIV-C acquisition when compared to controls, contrasting sharply with the results of the SIV-Alum^Alh12.5^ study [18] with 6.6-fold more alum salt (12.5:1 Alum:gp120) in what was an otherwise identical immunization regimen. Indeed, the second study (Alum^N10.8^; Study 2) performed here contained the same vaccine modalities used at a higher ratio of 10.8:1 Alum:gp120 and elicited V2-specific IgG that correlated with a decreased risk of Tier 1 SHIV-C acquisition. Mirroring the SIV-Alum^Alh12.5^ study, antibodies to V2 were revealed to be a correlate of decreased risk of SIV_mac251_ acquisition. The MF59 regimen of the present work (Study 3) induced the highest neutralizing antibodies titers to Tier 1 SHIV-C, correlated with plasma levels of C-Reactive Protein (CRP). The levels of V2 IgG were negligible in the mucosa of the animals immunized in this regimen.

*In vitro* stimulation of human and macaque PBMCs demonstrated that decreasing the dose of Alum affects the production of IL-1β. Vitally, this cytokine induces innate monocyte memory: a response that strongly correlated with a reduced risk of acquisition of the neutralization-resistant Tier 2 SIV_mac251_ [17]. Thus, the differing vaccine efficacies of the SIV_mac251_ and SHIV-C macaque models might depend in part on the inflammatory milieu generated by the combination of the vectored vaccine and adjuvant, and the neutralization profile of the challenge virus in each regimen. More specifically, these data suggest that low doses of Alum salt may be less effective in harnessing monocyte innate memory via IL-1β induction, thereby limiting the efficacy of Alum-based vaccine regimens.

We demonstrate here that the MF59 regimen protects against the easily-neutralized Tier 1 SHIV-C. In prior work, however, an MF59 regimen based on the same vaccine modalities did not protect against the neutralization-resistant Tier 2 SIV_mac251_ [18]. Given that 99% of the circulating HIV variants in South Africa have a neutralization-resistant profile, it is uncertain whether the Tier 1 SHIV-C model will be predictive of vaccine efficacy in the HVTN702 HIV trial. Ultimately, we must await the results of HVTN702 to gauge the accuracy of predictions based on the SIV_mac251_ or SHIV-C macaque models.

## Results

### Alum^N1.9^ IgA to V2 do not reduce Tier 1 SHIV-C acquisition

**Fig 1:**
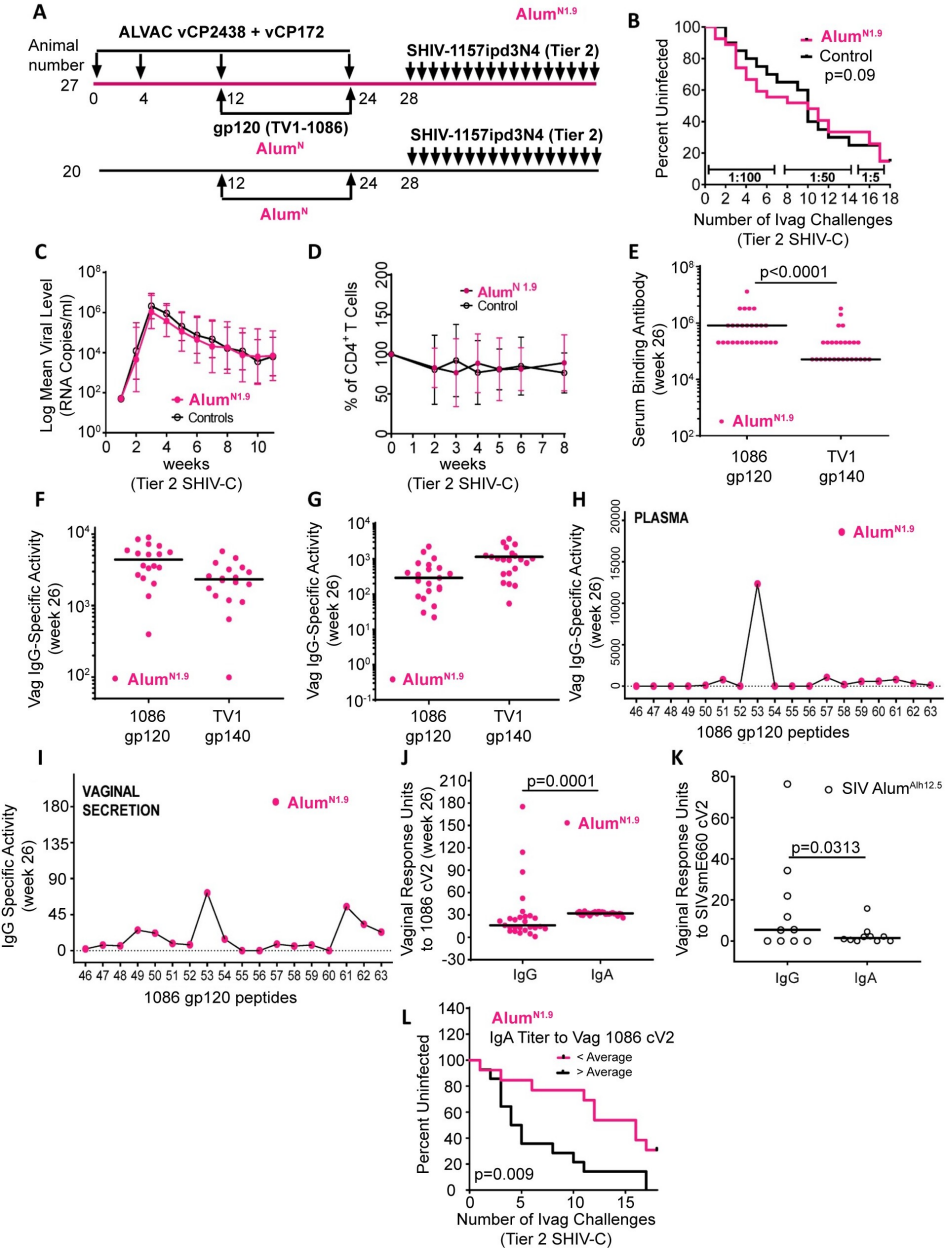
Relative efficacy and immunogenicity of the Alum^N1.9^ regimen. Unless stated otherwise, all assays, experiments and graphs associated with the Alum^N1.9^ study are represented by full red circles, and available samples (i.e., those not depleted in other experiments) are shown as stated in the legend. (A) Animals were immunized with ALVAC-vCP172 and ALVAC-cVCP2438 and boosted twice with monomeric gp120 proteins (200 μg each of TV1 and 1086) in 0.75 mg doses of Aluminum hydroxide manufactured by Novartis (Alum^N1.9^; n = 27; Table 1). Controls (n = 20 received only two Alum^N^ adjuvant inoculation at the same dose as the immunized macaques. (B) The rate per exposure of Tier 2 SHIV-C (SHIV-1157ipd3N4) acquisition was compared in 27 vaccinated animals and 20 controls using the log-rank test of the discrete-time proportional hazards model. (C) Geometric mean of SHIV-C RNA levels in plasma of the 23 vaccinated and 17 control animals that became infected. (D) Mean percentage of CD4^+^ T cell changes in blood (after infection in 23 vaccinated and 17 control animals. (E) Serum reactivity to the entire 1086 gp120 and TV1 gp140 at week 26 (two weeks after final immunization) in all 27 immunized animals (Mann-Whitney test). (F) Vaginal IgG-specific activity to the entire 1086 gp120 and TV1 gp140 in 18 of 27 vaccinated animals at week 26 (measured as MFI × dilution.total IgG [μg/ml]). (G) Vaginal IgG-specific activity to 1086 and TV1 V1/V2 scaffolds in 21 of 27 vaccinated animals at week 26. (H) Mean plasma IgG specific-activity to the 18 overlapping peptides encompassing 1086 gp120 V2 in all 27 vaccinated animals at week 26. Two animals did not exhibit V2 reactivity (Table 2 and S1B Fig). (I) Mean of vaginal IgG specific-activity to the 18 overlapping peptides encompassing 1086 gp120 V2 at week 26 in 13 immunized macaques. Three of the 13 animals analyzed did not demonstrate V2 reactivity (Table 2 and S1B Fig). (J) Vaginal IgG and IgA response units to 1086 cV2 in all 27 immunized macaques. (K) Vaginal IgG and IgA response units to SIV_smE660_ cV2 in the 10 females (out of 27 total rhesus macaques) immunized in the SIV-Alum^Alh^ study (open black circles). (L) Rate of Tier2 SHIV-C acquisition in the 27 animals vaccinated with Alum^N1.9^. Animals with above-average (black, 14 animals) and below-average (pink, 13 animals) IgA titers to 1086 cV2 in vaginal mucosa.

In the Alum^N1.9^ study (Study 1), a group of 27 macaques was administered four doses of ALVAC-SIV *gag-pol* (vCP172) and ALVAC-HIV *gag-pro-env* (vCP2438) at weeks 0, 4, 12, and 24. At weeks 12 and 24, all 27 macaques received 200 μg each of clade C TV1 and 1086 gp120 proteins (for a total of 400 μg/dose) formulated in 0.75 mg of proprietary Alum^N1.9^ (1.9:1 Alum:gp120; Fig 1A; Table 1). SIV-Alum^Alh12.5^ was previously protective against SIV_mac251_ using a boost of 800 μg of gp120 formulated with 5.0 mg of Alum^Alh12.5^ (12.5:1 Alum:gp120; Table 1) [18].

**Table 1:** Adjuvant and gp120 envelope protein dosage and correlates of risk of SHIV-C acquisition.

Study	Alum salt/dose (mg)	Total protein boosts	Total Alum salt (mg/animal)	gp120 dose (total mg)	Alum salt/gp120 (ratio)	Vaccine Efficacy (%) / Risk of acquisition
**Alum**^**N1.9**^ **Tier 2** **(SHIV-C)**	0.750	2	1.5	2 × 0.4 = **0.8**	1.9	None/ **IgA to V2** Increased risk
**Alum**^**N10.8**^ **Tier 1** **(SHIV-C)**	2.16	4	8.6	4 × 0.2 = **0.8**	10.8	None/ **IgG to V2** Decreased risk
**MF59** **Tier 1** **(SHIV-C)**	N/A	4	N/A	4 × 0.2 = **0.8**	N/A	(64%)/ **Neutralizing Ab titers** Decreased risk
**Alum**^**Alh12.5**^ **Tier 2** **(SIV**_**mac251**_**)**	5	2	10	2 × 0.4 = **0.8**	12.5	(44%)/ **IgG to V2** decreased risk
**MF59** **Tier 2** **(SIV**_**mac251**_**)**	N/A	2	N/A	2 × 0.4 = **0.8**	N/A	None/ **IgG to V2** Increased risk

Amount of adjuvant and gp120 protein used in the current SHIV-C and previously published SIV_mac251_ macaque studies [18].

Vaccine efficacy was assessed as the average rate of virus acquisition in the 27 vaccinated animals versus 20 controls that received only two inoculations of Alum^N^ following a total of 17 weekly intravaginal challenges with the Tier 2 SHIV-C (SHIV-1157ipd3N4) [20], starting 4 weeks after the last immunization (Fig 1A). Compared to controls, Alum^N1.9^ did not reduce the risk of acquisition of Tier 2 SHIV-C (Fig 1B), and no evidence of viral control was observed (Fig 1C). Likewise, neither the vaccinated nor the control groups experienced a significant decrease in CD4^+^ T cells, indicative of the limited pathogenic profile of this Tier 2 SHIV-C (Fig 1D). The lack of vaccine efficacy was somewhat surprising, as we had previously observed 44% efficacy with SIV-Alum^Alh12.5^. Noting this discrepancy, we analyzed systemic and vaginal antibody responses and contrasted them with those obtained in the SIV-Alum^Alh12.5^ study. Following the final immunization of Alum^N1.9^, serum binding antibody titers to 1086 (av. reactivity 10^6^) were significantly higher than those to TV1 (av. reactivity 10^5^; *P <* 0.0001; Fig 1E). Vaginal IgGs specific for the two envelope proteins (Fig 1F) or the gp70 V1/V2 scaffolds (Fig 1G) did not differ between antigens and were similar to levels observed in the SIV-Alum^Alh12.5^ study [18].

We further characterized V2-specific IgG levels in plasma and vaginal secretions by performing peptide arrays of overlapping peptides encompassing the 1086 V2 region (S1A Fig). Of the 27 animals vaccinated with Alum^N1.9^, 25 (92.5%) had IgG in blood that recognized peptide 53 (TELKDKKHKVHALFY) containing the cryptic α_4_β_7_ binding site (Fig 1H). Peptide 57 (LFYKLDVVPLNGNSS), containing the canonical tripeptide α_4_β_7_ integrin receptor binding site, was recognized by the IgG of only four of these animals (S1B and S1D Fig and S2 Fig). Measured in vaginal secretions, IgGs recognizing peptide 53 were present in 10 of the 13 animals tested (78%; Fig 1I; Table 2; S1C and S1E Fig).

**Table 2:** Frequency of systemic and mucosal IgG recognition of V2 peptide 53.

Study	Alum^N1.9^	Alum^N10.8^	MF59
**Plasma**	92.5% (25/27)	100% (9/9)	100% (9/9)
**Vaginal secretion**	78% (10/13)	33% (1/9)	0% (0/9)

Frequency of animals with detectable 1086 peptide 53 (TELKKHKVAHALFY) specific IgG in plasma or vaginal secretion.

An important qualitative difference between the current Alum^N1.9^ study and SIV-Alum^Alh12.5^ was observed in the levels of mucosal V2-specific IgA and IgG. In Alum^N1.9^, V2-specific IgA was higher than IgG (*P* = 0.0001; Fig 1J). In stark contrast, the level of SIV V2-specific IgG was significantly higher than V2-specific IgA (*P* = 0.0313; Fig 1K) in the females of SIV-Alum^Alh12.5^. Importantly, correlate of risk analyses of all antibody responses in Alum^N1.9^ measured above revealed that the level of vaginal IgA correlated with earlier acquisition of Tier 2 SHIV-C (*P* = 0.009; Fig 1L). Notably, the mucosal IgG level was a primary correlate of decreased risk of SIV_mac251_ acquisition in the SIV-Alum^Alh12.5^ study [18], and high serum IgA was a correlate of increased HIV risk in RV144 [21]. While Alum^N1.9^ induced neutralizing antibodies to Tier 1A HIVs and moderately to SHIV-1157ipEL-p [22], antibodies against Tier 2 HIVs or SHIV-1157ipd3N4 were not produced (Table 3). None of the neutralization titers correlated with Tier 2 SHIV acquisition.

**Table 3:** Serum neutralizing activity in animals immunized in Study 1 (Alum^N1.9^).

Alum^N1.9^	NEUTRALIZATION—ID50 in TZM-bl Cells Pseudoviruses produced in 293T cells					Challenge Virus	
	MW965.26 Tier 1A Clade C	MN.3 Tier 1A Clade B	TV1.21 Tier 1B Clade C	Ce1086_B2 Tier 2 Clade C	96ZM651 Tier 2 Clade C	SHIV- 1157ipEL-p Tier 1	SHIV-1157ipd3N4 Tier 2
Animal ID	week 26	week 26	week 26	week 26	week 26	week 28	week 28
MEC	1198	57	< 20	< 20	< 20	26	21
MGM	1512	83	< 20	< 20	< 20	22	< 20
MGT	1849	41	< 20	< 20	< 20	26	< 20
MHF	11061	83	< 20	< 20	< 20	55	< 20
MHZ	3945	40	< 20	< 20	< 20	37	< 20
MKF	1564	104	< 20	< 20	< 20	21	< 20
MJZ	2177	229	< 20	< 20	< 20	29	< 20
MIV	1390	58	< 20	< 20	< 20	25	< 20
MIR	1227	121	< 20	< 20	< 20	21	< 20
MIH	2164	52	< 20	< 20	< 20	23	< 20
KNH	2265	191	< 20	< 20	< 20	31	< 20
KI9	1372	145	< 20	< 20	< 20	33	< 20
DL5A	1767	68	< 20	< 20	< 20	28	25
CW9M	977	105	< 20	< 20	< 20	21	< 20
DN6W	488	33	< 20	< 20	< 20	25	20
MCX	2525	207	< 20	< 20	< 20	31	< 20
MBD	2160	183	< 20	< 20	< 20	22	< 20
MDN	2679	354	< 20	< 20	< 20	–	–
DM7R	717	92	< 20	< 20	< 20	24	< 20
KIJ	1959	933	< 20	< 20	< 20	25	< 20
MKK	321	55	< 20	< 20	< 20	20	< 20
MR4	779	137	< 20	< 20	< 20	30	< 20
MT3	641	< 20	< 20	< 20	< 20	21	< 20
MZO	1081	52	< 20	< 20	< 20	22	< 20
PME	1168	79	< 20	< 20	< 20	< 20	< 20
PZZX	2229	37	< 20	< 20	< 20	23	< 20
PZC	1383	475	< 20	< 20	< 20	21	< 20

Serum neutralizing antibody activity in Alum^N1.9^.

### Alum^N10.8^ IgG to V2 and delayed Tier 1 SHIV-C acquisition

**Fig 2:**
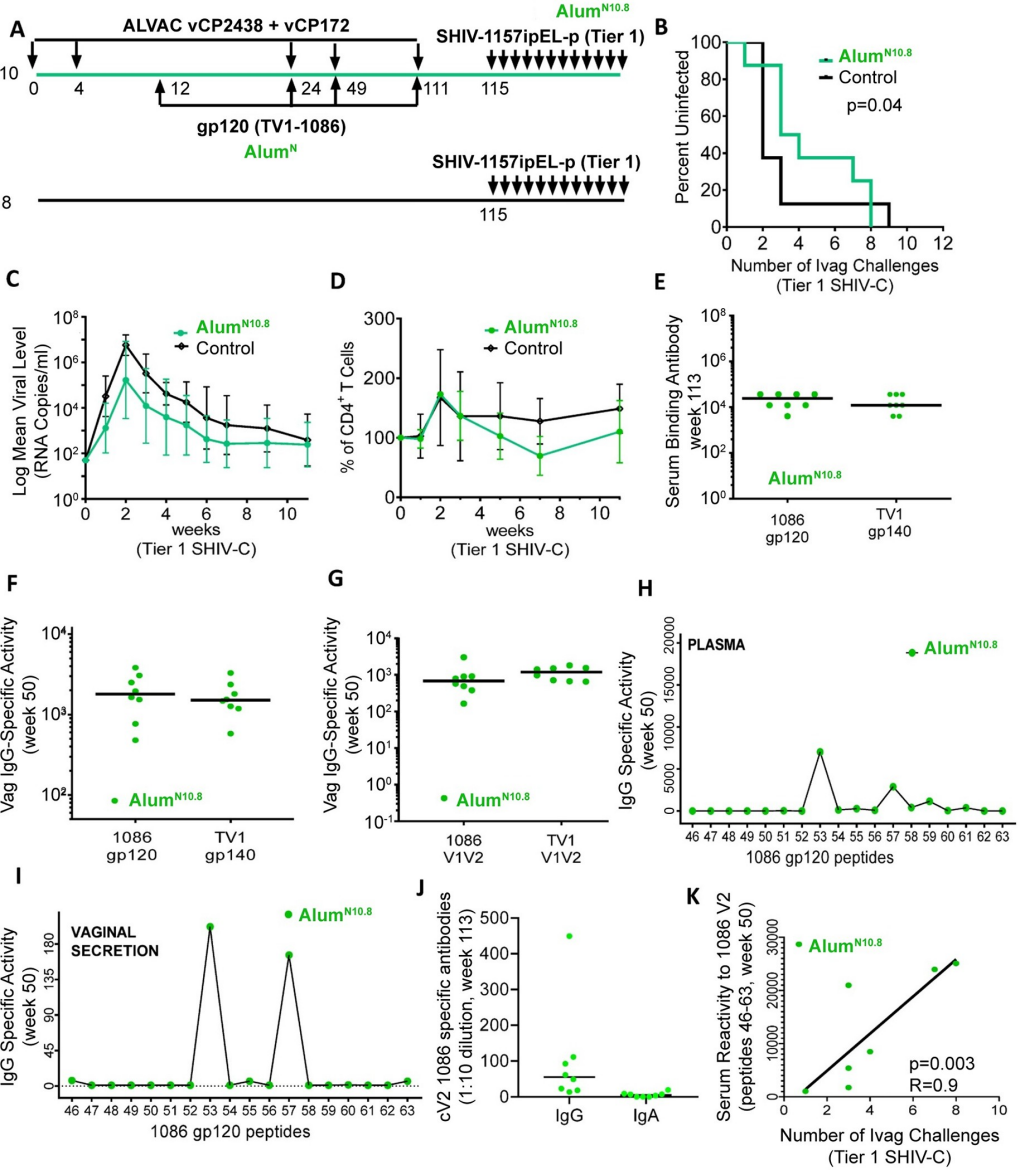
Relative efficacy and immunogenicity of the Alum^N10.8^ regimen. Unless stated otherwise, all assays, experiments and graphs associated with the Alum^N10.8^ study are represented by full green circles, and the number of available samples (i.e., those not depleted in other experiments) are shown as stated in the legend. (A) Animals were immunized with ALVAC-vCP172 and ALVAC-cVCP2438 and boosted 4 times with 100 μg each of the monomeric TV1 and 1086 gp120 proteins (800 μg total). Each dose was formulated with 2.16 mg of Aluminum Hydroxide (Alum^N10.8^; Table 1). Three of four boosts were administered together with the ALVAC vectors (Alum^N10.8^; n = 10). Only 8 out of 10 vaccinated animals survived up to the Tier 1 SHIV-C challenge. Controls (n = 8; were left naïve. (B) The rate per exposure of Tier 1 SHIV-C (SHIV-1157ipEL-p) acquisition was compared between 8 vaccinated animals and 8 controls using the log-rank test of the discrete-time proportional hazards model. (C) Geometric mean of SHIV-C RNA levels in plasma of 8 vaccinated and 8 control animals that became infected. (D) Mean percentage of CD4^+^ T cell changes in blood (after infection in 8 vaccinated and 8 control animals. (E) Serum reactivity to 1086 gp120 and TV1 gp140 in 8 vaccinated animals at week 113 (two weeks after last immunization). (F) Vaginal IgG-specific activity to 1086 gp120 and TV1 gp140 in 8 vaccinated animals at week 50. (G) Vaginal IgG-specific activity to 1086 and TV1 V1/V2 scaffolds in 8 vaccinated animals at week 50. Mean IgG specific-activity to the 18 overlapping linear peptides encompassing the 1086 V2 measured in the (H) plasma or (I) vaginal secretions of 9 vaccinated animals at week 50 (see S3A Fig for data from each animal). (J) Cyclic V2 1086 specific IgG and IgA levels in vaginal secretions collected from immunized macaques two weeks before challenge exposure (week 113). (K) Spearman correlation of the sum of serum reactivity to 1086 V2 (peptides 43–63) at week 50 and the week of intravaginal acquisition of Tier 1 SHIV-C (SHIV-1157ipEL-p) in the 8 immunized animals of the Alum^N10.8^ group.

We next tested Alum^N10.8^ (Study 2) in 10 Chinese rhesus macaques in a longer immunization regimen. Animals were vaccinated at weeks 0, 4, 24, 49, and 111 with ALVAC-SIV *gag-pol* (vCP172) and ALVAC-HIV *gag-pro-env* (vCP2438), and at weeks 12, 24, 49, and 111 with 100 μg each of clade C TV1 and 1086 proteins (for a total of 200 μg per dose) formulated in 2.16 mg of Alum^N^ (Alum salt:gp120 10.8:1; Alum^N10.8^; Fig 2A; Table 1). Two of the original 10 animals in both Alum^N10.8^ and MF59 groups, for a total of 4 animals, perished due to health conditions unrelated to the study. As a result, only 8 immunized animals per group were exposed to SHIV-C challenge together with 8 additional naïve controls. Twelve challenges with the neutralization-sensitive Tier 1 SHIV-1157ipEL-p were performed intravaginally four weeks after the last immunization in all these animals. We observed no significant reduction in the risk of SHIV-C acquisition (*P* = 0.4; Fig 2B) and only a transient decrease in plasma virus levels (at week 2) when compared to controls (*P* = 0.010; Fig 2C). Consistent with the low pathogenic profile of this virus, no significant decrease in CD4^+^ T cells was observed when compared to the baseline levels in either the vaccinated or control groups (Fig 2D).

Analysis of humoral immune responses demonstrated that Alum^N10.8^ induced equivalent serum envelope binding antibody titers to 1086 and TV1 (Fig 2E) in Alum^N10.8^, but at lower levels than Alum^N1.9^ (Fig 1E). Despite divergence here, vaginal IgG levels to the 1086 and TV1 envelope proteins or the corresponding V1/V2 scaffolds in Alum^N10.8^ and Alum^N1.9^ were comparable (Fig 2F and 2G). IgG specific for 1086 V2 peptide 53 was detected in the plasma of all animals, but it was only found in the vaginal secretions of 33% of animals immunized with Alum^N10.8^ (Fig 2H, Table 2). Only one animal was found to have IgG to peptide 57 (Fig 2I; Data on all animals are presented in S3A Fig). Interestingly, the increase in the alum dose in this vaccinated group did result in an inversion of the proportion of cyclic V2 1086 specific IgG and IgA that we had observed in the Alum ^N1.9^ study (compare Figs 2J to 1J). The level of these two V2 specific vaginal responses did not correlate with the risk of SHIV-C acquisition.

Even though this vaccination regimen did not significantly decrease the risk of Tier 1 SHIV-C acquisition compared to controls, correlate of risk analysis demonstrated that serum antibody levels to V2 strongly correlated with reduced risk of SHIV-C acquisition (R = 0.9, *P* = 0.03; Fig 2K). This regimen did induce neutralizing antibodies to Tier 1 HIVs and SHIV-1157ipEL-p, but not to Tier 2 HIVs or SHIV-1157ipd3N4 (Table 4). However, the neutralization titers did not correlate significantly with the time of in the 8 vaccinated animals exposed to Tier 1 SHIV-C challenge.

**Table 4:** Serum neutralizing activity in animals immunized in Study 2 (Alum^N10.8^).

Alum^N10.8^	Neutralization in TZM-bl cells, Pseudoviruses produced in 293T cells						Challenge Virus			
	MW965.26 Tier 1A Clade C*	MW965.26 Tier 1A Clade C*	MN.3 Tier 1A Clade B*	TV1.21 Tier 1B Clade C	Ce1086_B2 Tier 2 Clade C	96ZM651 Tier 2 Clade C	SHIV-1157ipEL-p Tier 1	SHIV-1157ipEL-p Tier 1	SHIV-1157ipd3N4 Tier 2	SHIV-1157ipd3N4 Tier 2
Animal ID	week 50	week 115	week 50	week 50	week 50	week 50	week 50	week 115	week 50	week 115
**416**	3306	864	24.5	10	10	10	40	46	10	10
**4600**	3677	–	583.5	10	10	10	30	–	10	–
**4624**	1327	639	31	10	10	**24**	20	31	10	10
**4997**	–	–	–	–	–	–	–	–	–	–
**5256**	3787	501	476	10	10	10	22	32	10	10
**20012**	559.5	152	36	10	10	10	20	10	10	10
**20240**	1897	174	30	10	10	10	20	22	10	10
**20247**	463	377	145.5	10	10	10	20	20	10	10
**00R2240**	2986	627	248	10	10	10	20	21	10	10
**98R0724**	9272	3985	1414	10	10	10	42	85	10	10

Serum neutralizing antibody activity in Alum^N10.8^.

*Average of two independent reports

### MF59 reduced risk of Tier 1 SHIV-C acquisition correlates with homologous neutralization

**Fig 3:**
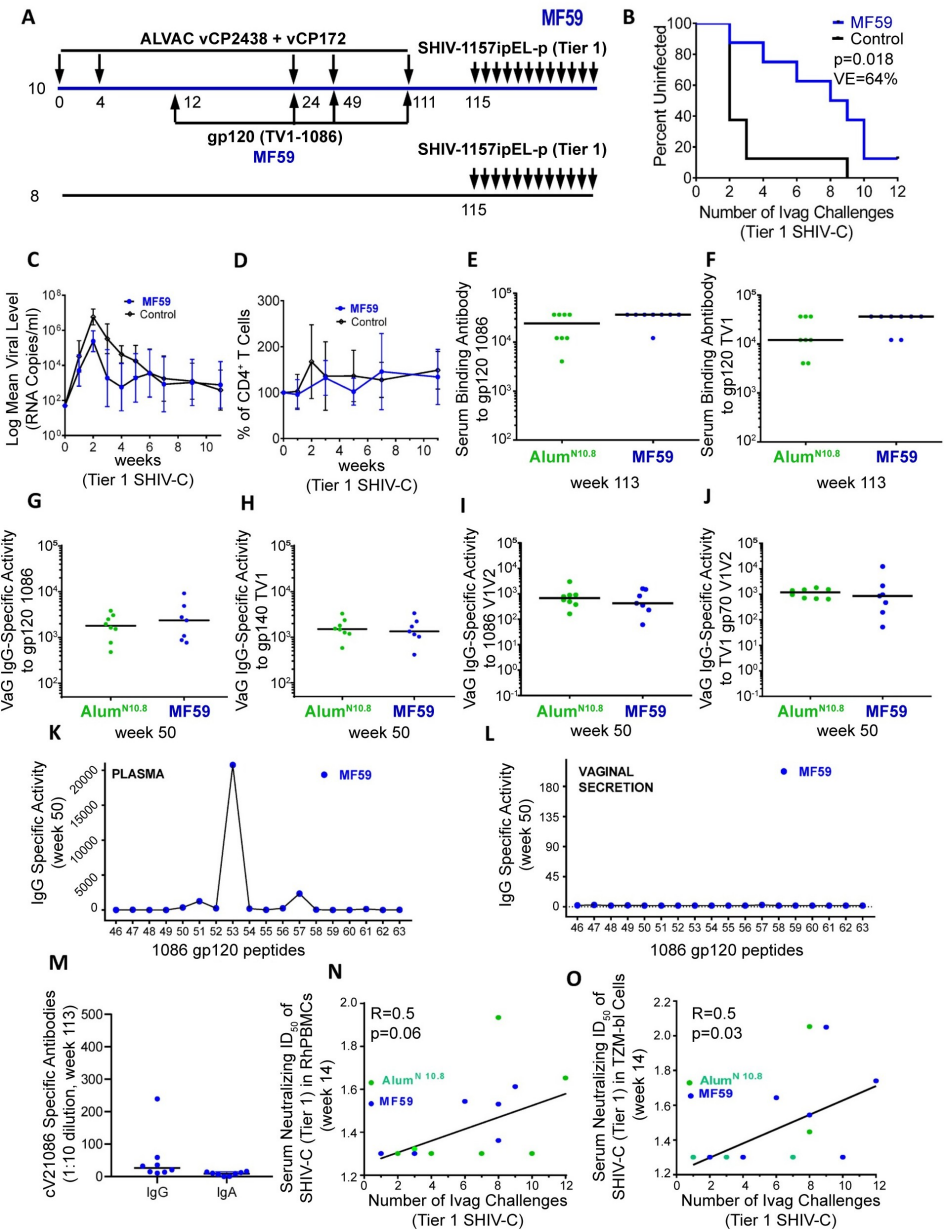
Relative efficacy and immunogenicity of the MF59 regimen. Unless stated otherwise, all assays, experiments and graphs associated with the MF59 study are represented by full blue circles, those of the Alum^N10.8^ study are represented by full green circles, and only available samples (i.e., those not depleted in other experiments) are shown. The number of samples represented in each graph is stated below. (A) Animals were immunized using ALVAC-vCP172 and ALVAC-cVCP2438 and boosted 4 times with 100 μg each of the monomeric TV1 and 1086 gp120 proteins (800 μg total). Each dose was formulated with MF59. Only 8 out of the 10 vaccinated animals survived to the time of challenge (simultaneous with the challenge of the Alum^N10.8^ group and naïve controls). (B) The rate per exposure of Tier 1 SHIV-C (SHIV-1157ipEL-p) acquisition was compared in 8 vaccinated animals and 8 controls using the log-rank test of the discrete-time proportional hazards model. (C) Geometric mean of SHIV-C RNA levels in plasma of 7 vaccinated and 8 control animals that became infected. (D) Mean percentage of CD4^+^ T cell changes in blood (after infection in 7 vaccinated and 8 control animals). Serum binding antibodies to (E) gp120 1086 and (F) gp120 TV1 in 8 of 10 animals each in the Alum^N10.8^ and MF59 groups two weeks before intravaginal Tier 1 SHIV challenges. Vaginal IgG-specific activity to (G) gp120 1086 and (H) gp140 TV1 in 8 of 10 animals in both Alum^N10.8^eand MF59 at week 50. Vaginal IgG-specific activity to the V1/V2 (I) 1086 or (J) TV1 scaffold in 8 of 10 animals in both Alum^N10.8^ and MF59 at week 50. (K) Plasma or (L) vaginal IgG reactivity (mean value) to the 18 overlapping linear peptides encompassing the 1086 V2 in nine MF59-vaccinated animals at week 50. All 9 vaccinated animals that survived up to week 50 had reactivity to V2 peptides in plasma, but not in the vaginal secretions (see S3B Fig for data on all animals). (M) Cyclic V2 1086 specific IgG and IgA levels in vaginal secretions collected from immunized macaques two weeks before challenge exposure (week 113). (N, O) Spearman correlations between levels of serum antibodies (week 14) from the 18 immunized animals in both groups capable of neutralizing the Tier 1 SHIV-C (SHIV-1157ipEL-p) challenge stock in rhesus (N) PBMCs or (O) TZM-bl cells and the week of intravaginal SHIV-1157ipEL-p acquisition.

The final group in this work was adjuvanted with MF59 (Study 3). This study was performed simultaneously with Study 2 in the same animal facility. Ten Chinese rhesus macaques were immunized at weeks 0, 4, 24, 49, and 111 with ALVAC-SIV *gag-pol* (vCP172) and ALVAC-HIV *gag-pro-env* (vCP2438), and at weeks 12, 24, 49 and 111 with 100 μg each of clade C TV1 and 1086 proteins formulated with MF59 as summarized in Fig 3A and Table 1. Like those of the Alum^N10.8^ group, the animals in Study 3 were exposed to 12 doses of Tier 1 SHIV-1157ipEL-p by the intravaginal route. Both groups shared the same 8 naïve controls. The MF59 vaccine regimen significantly decreased the risk of Tier 1 SHIV-C acquisition compared to the controls (*P* = 0.018, log-rank test), showing an estimated vaccine efficacy of 64% at each challenge (Fig 3B). This is a notable contrast from the SIV-MF59 regimen that did not decrease the acquisition risk of the neutralization-resistant Tier 2 SIV_mac251_ [18]. In the current study, we observed a transient decrease of plasma virus (week 2) in immunized animals compared to controls (*P* = 0.0022, Wilcoxon rank-sum test; Fig 3C). No significant decrease in CD4^+^ T cells was observed between MF59 immunized animals and controls when compared to baseline levels (Fig 3D).

No discernable difference in serum antibody titers to 1086 or TV1 was observed between the MF59 and Alum^N10.8^ groups (Fig 3E and 3F). HIV-specific IgG levels in vaginal secretions were similar to those observed in Studies 1 and 2 for both the 1086 and TV1 gp120s (Fig 3G and 3H) and their corresponding gp70 V1/V2 scaffolds (Fig 3I and 3J). While V2-specific IgG was observed in the sera of all animals (Fig 3K), they were not detected in vaginal secretions (Fig 3L; Table 2; S3B Fig). The contrast between serum and mucosal responses to V2 was also observed in SIV-MF59 and SIV-Alum^Alh^ (see Fig 3C and 3D in ref [18]). No difference was observed between cyclic V2 1086 specific IgG and IgA in vaginal secretions obtained two weeks before challenge (week 113; Fig 3M), and these responses did not correlate with the risk of SHIV-C acquisition.

Neutralizing antibody titers to the Tier 1 HIV and SHIV-1157ipEL-p challenge viruses or the challenge stock in macaque PBMCs and TZM cells were generally higher in animals immunized with MF59 than Alum^N1.9^ (Tables 4 and 5) as expected [23–25]. The MF59 regimen did not induce neutralizing antibodies to Tier 2 HIVs (Table 5).

**Table 5:** Serum neutralizing activity against HIV strains in animals immunized in the MF59 study.

MF59	Neutralization in TZM-bl cells, Pseudoviruses produced in 293T cells					
	MW965.26 Tier 1A Clade C*	MW965.26 Tier 1A Clade C*	MN.3 Tier 1A Clade B*	TV1.21 Tier 1B Clade C	Ce1086_B2 Tier 2 Clade C	96ZM651 Tier 2 Clade C
Animal ID	week 50	week 115	week 50	week 50	week 50	week 50
**2316**	1226	1581	68.5	10	10	10
**4640**	9004.5	2266	2590.5	10	10	10
**5247**	7163	1034	255.5	33	10	10
**20024**	4091.5	–	78	32	10	21
**20052**	–	–	–	–	–	–
**20094**	764.5	257	33	20	10	10
**20100**	676.5	554	16	10	10	10
**00R0960**	5830.5	1462	413.5	30	10	10
**00R1348**	3471	863	367	10	10	10
**01R0728**	4055.5	1694	10	10	10	10

Serum neutralizing antibody activity in Alum^N10.8^.

*Average of two independent reports

Interestingly, correlate of risk analysis demonstrated that neutralizing antibodies to Tier 1 SHIV-1157ipEL-p measured in macaque PBMCs or TZM-bl cells at week 14 (Tables 6 and 7) correlated with a decreased risk of Tier 1 SHIV-C acquisition (R = 0.5, *P* = 0.06 and R = 0.5, *P* = 0.03, respectively; Fig 3N and 3O).

**Table 6:** Serum neutralizing activity against SHIV-C challenge stocks in animals immunized in Study 2 (Alum^N10.8^).

Alum^N10.8^	Challenge Virus						
	SHIV- 1157ipEL-p	SHIV- 1157ipEL-p	SHIV- 1157ipEL-p	SHIV- 1157ipd3N4	SHIV- 1157ipEL-p	SHIV- 1157ipEL-p	SHIV- 1157ipd3N4
	Tier 1	Tier 1	Tier 1	Tier 2	Tier 1	Tier 1	Tier 2
	RhPBMCs	TZM-bl cells	TZM-bl cells	TZM-bl cells	TZM-bl cells	RhPBMCs	TZM-bl cells
Animal ID	week 14	week 14	week 50	week 50	week 115	week 115	week 115
**416**	86	113	40	10	46	46	10
**4600**	–	–	30	10	–	–	–
**4624**	20	20	20	10	31	31	10
**4997**	–	–	–	–	–	–	–
**5256**	21	20	22	10	32	32	10
**20012**	20	20	20	10	10	20	10
**20240**	21	20	20	10	22	22	10
**20247**	20	20	20	10	20	20	10
**00R2240**	23	28	20	10	21	21	10
**98R0724**	20	20	42	10	85	85	10

Serum neutralizing antibody activity against the Tier 1 and Tier 2 challenge stocks in Alum^N10.8^.

**Table 7:** Serum neutralizing activity against SHIV-C challenge stocks in animals immunized in Study 3 (MF59).

MF59	Challenge Virus						
	SHIV- 1157ipEL-p	SHIV- 1157ipEL-p	SHIV- 1157ipEL-p	SHIV- 1157ipEL-p	SHIV- 1157ipd3N4	SHIV- 1157ipEL-p	SHIV- 1157ipd3N4
	Tier 1	Tier 1	Tier 1	Tier 1	Tier 2	Tier 1	Tier 2
	RhPBMCs	TZM-bl cells	TZM-bl cells	TZM-bl cells	TZM-bl cells	RhPBMCs	TZM-bl cells
Animal ID	week 14	week 14	week 50	week 115	week 115	week 115	week 50
**2316**	20	20	21	30	10	30	10
**4640**	20	20	36	67	10	67	10
**5247**	35	44	50	24	10	24	10
**20024**	–	–	55	–	10	–	10
**20052**	–	–	–	–	–	–	–
**20094**	41	112	20	33	10	33	10
**20100**	34	35	21	43	10	43	10
**00R0960**	45	55	39	89	10	89	10
**00R1348**	20	20	24	75	10	75	10
**01R0728**	20	20	26	44	10	20	10

Serum neutralizing antibody activity against the Tier 1 and Tier 2 challenge stocks in MF59.

### MF59 inflammatory profile impacts neutralizing Abs

**Fig 4:**
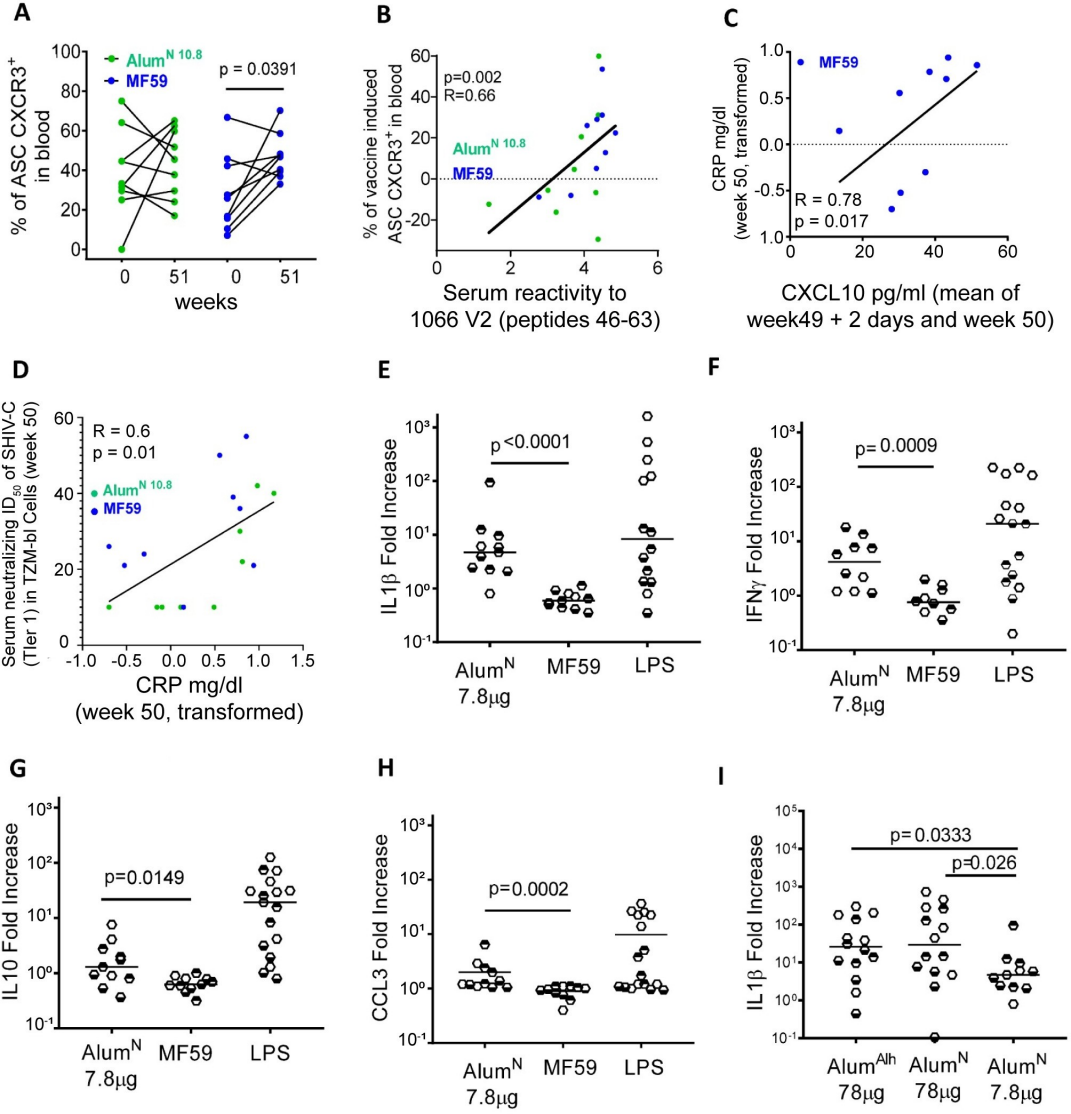
Inflammation parameters in immunized macaques. The vaccine-induced cells were calculated as the delta of weeks 51 and week 0. ASCs were defined as live cells, lineage- (CD3^-^ / CD14^-^ / CD16^-^ / CD56^+^) / (CD19^+^ / CD20^+^ / (CD21^-^CD27^+^) / Ki67^+/++^ / (CD38^+/++^ / CD39^+^). (A) Frequency of blood circulating ASCs expressing CXCR3 on their membrane in Alum^N10.8^ (green filled circles) and MF59 (blue filled circles) before (week 0) and after immunization (week 51). (B) Spearman correlation between the sum of the serum reactivity to the 18 linear peptides encompassing the 1086 V2 (peptides 43–63) and frequency of vaccine-induced, blood-circulating CXCR3^+^ASCs in Alum^N10.8^ and MF59 at week 51. The linear regression and *P* and R values were obtained using all 9 animals of the Alum^N10.8^ and MF59 groups. (C) Correlation between the levels of CRP (log transformed values) measured at week 50 and the mean of CXCL10 levels in serum measured in duplicate experiments (5 days apart: week 49 + 2 days, and week 50) in the MF59 group. (D) Correlation between serum neutralizing antibodies to SHIV Tier 1 CS in TZM-bl cells and C-Reactive Protein (log transformed-values) levels in all animals (9 animal each) of both Alum^N10.8^ and MF59. The linear regression and *P* and R values are derived from all 18 animals. (E) IL-1β, (F) IFN-γ, (G) IL-10, and (H) CCL3 levels measured in human (empty hexagons), Indian rhesus macaque (dark-top hexagons), and Chinese rhesus macaque (dark-bottom hexagons) PBMCs after stimulation with Alum^N^, MF59, and LPS as positive control in all panels. (I) IL-1β levels measured in human (empty circles), Indian rhesus macaque (dark-top hexagons), and Chinese rhesus macaque (dark-bottom hexagons) PBMCs after stimulation with Alum^Alh^ (78 μg) and Alum^N^ at the same concentration (78 μg) or in the same proportion (7.8 μg) as used *in vivo*. Horizontal lines represent the median values in all panels.

The different V2 mucosal and systemic responses observed in Alum^N10.8^ and MF59 raised the hypothesis that the two adjuvants may affect the homing of antibody-secreting cells (ASC). We therefore investigated the expression of gut homing marker α_4_β_7_ and CXCR3^+^ on the surface of ASCs as previously described [18]. We found that neither the α_4_β_7_^+^ nor CXCR3^+^ ASC subsets in the two groups differed significantly after immunization, but ASCs expressing CXCR3^+^ were significantly increased in the MF59 group post-vaccination versus the baseline (Fig 4A). In the MF59 group, the frequency of vaccine-induced CXCR3^+^ ASCs positively correlated with the serum level of antibodies to the overlapping peptides of 1086 V2 (R = 0.6, *P* = 0.002; Fig 4B).

These data suggest that MF59 might have created an inflammatory milieu that favored ASC homing to inflammatory sites, rather than to mucosal sites. Since the natural ligand of CXCR3 is the CXCL10 chemokine induced by IFN-γ [26] and CRP levels increase during inflammation, we measured both in the blood of the immunized animals. Their levels did not differ between animals immunized with MF59 and Alum^N10.8^ (S4A and S4B Fig), but the levels of CRP and CXCL10 were directly correlated with each other (R = 0.78, *P* = 0.017; Fig 4C). Interestingly, the level of CRP correlated with the neutralizing antibody titers to Tier 1 SHIV-C elicited by the MF59 vaccine regimen (R = 0.6, *P* = 0.01; Fig 4D).

### IL-1β production *in vitro* dependent on dosage of Alum

Because the pharmacokinetics of drugs differ according to body mass [27], we adhered to allometric scaling to determine an Alum dosage in SIV-Alum^Alh12.5^ that would deliver comparable effects to those of Alum Alhydrogel elicited in larger animals (12.5:1 Alum:gp120; 6.6-fold the amount of Alum used in Alum^N1.9^; Table 1). We hypothesized that the lower dosage of the proprietary Alum^N^ used in the current study may have affected cytokine responses induced by vaccination. IL-1β in particular is induced by ALVAC via AIMS activation [28]. This cytokine provides the signals that direct innate monocyte memory responses, which have in turn been shown to correlate with the decreased risk of SIV_mac251_ acquisition afforded by this vaccine modality [17, 29].

Alum directly infects monocytes and activates the inflammasome, induces IL-1β [30], and harnesses innate monocyte responses [31, 32]. We studied the effects of these adjuvants on human and macaque PBMCs and found that *in vitro* stimulation with Alum^N^ induced significantly higher amounts of IL-1β (*P* < 0.0001), IFN-γ (*P* = 0.0009), IL-10 (*P* = 0.0149), and CCL3 (*P* = 0.0002) compared to MF59 (Fig 4E–4I), while both adjuvants induced similar levels of IL-13 (S4F and S4J Fig). Notably, Alum^Alh^ also induced significantly higher amounts of IL-1β (*P* < 0.0001), IFN-γ (*P* = 0.0004), IL-13 (*P* = 0.013) and CCL3 (*P* = 0.0002) compared to MF59 (S4D–S4G Fig). No significant differences were observed when comparing the effects of equivalent amounts of Alum^Alh^ and Alum^N^ (S4H–S4K Fig). Data parsed for human or macaque PBMCs demonstrated that human PBMCs are generally more responsive to Alum (S5 Fig).

**Fig 5:**
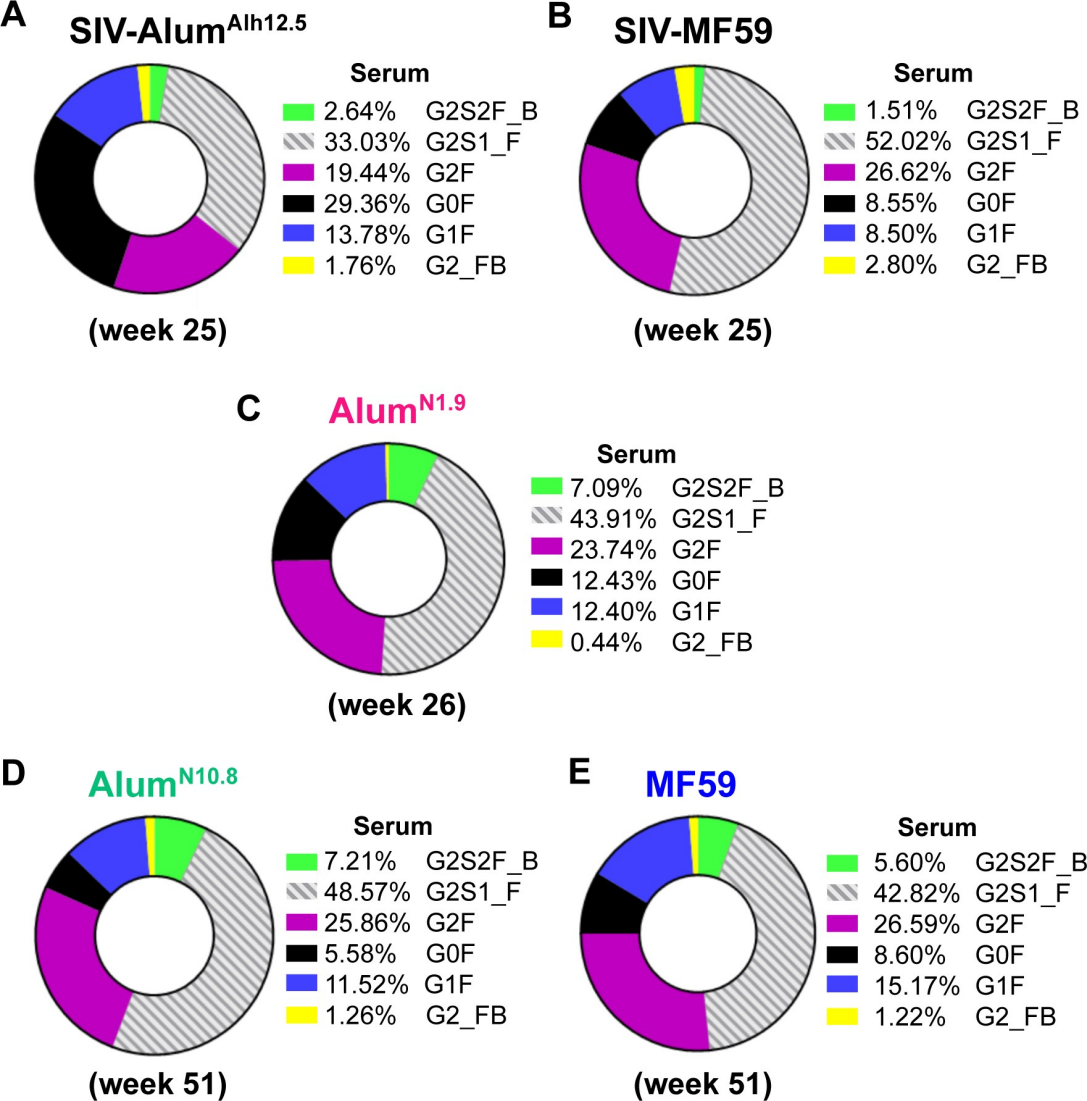
Anti-gp120 antibody glycoforms in serum of SIV and SHIV studies using MF59, Alum^Alh^, or Alum^N^ adjuvants. Anti-gp120 antibody glycoforms in vaccinated animals: (A) SIV-Alum^Alh12.5^, n = 27; (B) SIV-MF59, n = 27; (C) Alum^N1.9^, n = 27; (D) Alum^N10.8^, n = 8; (E) MF59, n = 8. The Fc-domain of antibodies can be classified by the presence or absence of different sugar group such as 1 fucose (“F”) group, 1 bisecting-n-acetyl glucosamine (“B”) group, up to 2 galactoses (“G0,” no galactose; “G1,” 1 galactose; “G2,” 2 galactose), or up to 2 sialic acids (“S1,” 1 sialic acid; “S2,” 2 sialic acids). Different sugar group patterns mediated or inhibited different antibody functions as described by Ackerman et al. [52].

In order to evaluate possible differences in antibody Fc-associated functions related to the macaque species or the quantity or quality of the adjuvant, we characterized the Fc profile of TV1 envelope-specific antibodies in the sera of the immunized animals at week 26 or 51. We previously found that the percentage of Fc with G0F, G1F, G2F, and G2S1F sugars differed significantly between Indian rhesus macaques immunized with SIV-Alum^Alh^ and those with SIV-MF59(Fig 5A) [17]. In Chinese rhesus macaques, we also found significant differences in G0F, G1F, G2F, and G2S1F between the Alum^N1.9^ and Alum^N10.8^ groups (Fig 5C–5E; S6 Fig). As in SIV-MF59, G2F was highest with the MF59 adjuvant used in Study 3. However, none of these gp120-specific antibody glycan Fc forms correlated with SHIV-C acquisition or the level of plasma viral RNA. The Fc functional profile did not correlate with CD4 counts or with anti-envelope binding or neutralizing antibodies, as was also observed in animals immunized with similar vaccine regimens and challenged with SIV_mac251_ [17].

## Discussion

The partial success of RV144 in preventing clade B/E HIV-1 infection is an important milestone, but it must be extended to include the clade C strains that account for nearly 50% of all new HIV-1 infections worldwide [33]. Analysis of RV144 suggests that vaccine efficacy was not durable using Alum-Alhydrogel (60% efficacy 12 months post-vaccination [34]; 31.2% after 42 months [6]). HVTN702 was thus designed to induce higher and more durable immune responses using the oil-in-water MF59 adjuvant. In the SIV_mac251_ macaque model, the use of different primes, immunogens, or adjuvants influenced the risk of SIV_mac251_ acquisition [17, 18, 35, 36] and allowed for the identification of novel correlates of decreased risk such as innate monocyte memory, NKp44 cells, and antibodies to V2 [17, 18]. In the same study, we also demonstrated that the Alum and MF59 adjuvants shaped immune responses differently, and even though SIV-MF59 was more immunogenic, it did not decrease the risk of SIV_mac251_ acquisition like SIV-Alum^Alh12.5^ did [18]. The present studies were conceived to assess the relative efficacy of the ALVAC-HIV Clade B/C regimen with the clade C envelope gp120 1086 and TV1 proteins as boost against SHIV-C challenge viruses with different neutralization profiles.

There were, however, constraints and limitations in the design of Study 1 related to the manufacturer’s guidelines in formulating the gp120 boosts. The Alum^N1.9^ regimen tested here mimicked the number and order of immunizations performed in RV144 and our SIV-Alum^Alh^ study, but while the gp120 boosts were maintained at the same dose as used in SIV-Alum^Alh^ and RV144 (200 μg each), a much lower dose of Alum^N^ was used in Study 1 (750 μg/dose). Although the FDA and CDC approve of no more than 850 μg of Alum Alhydrogel per dose in human vaccines [37, 38], we used 5,000 μg/dose in SIV-Alum^Alh^ to compensate for the well-documented pharmacokinetic differences among differently sized species [27]. Counterintuitively, this typically means that a drug must be administered at a higher dose in smaller animals such as macaques to obtain the same effects elicited in larger species. The Alum^N1.9^ and SIV-Alum^Alh12.5^ studies were both performed in Indian rhesus macaques, used equivalent vaccine modalities and regimens against SIVmac251, and were statistically powered to obtain a readout of vaccine efficacy (27 vaccinees and 20 controls in Alum^N1.9^), but despite their many similarities, we found that Alum^N1.9^ was not efficacious against the Tier 2 SHIV-C. Thus, the data suggest that the low dose of Alum might have contributed to the high level of mucosal V2-specific IgA that correlated with an increased risk of virus acquisition. MF59 was not made available by the manufacturer for testing in a parallel study against the Tier 2 SHIV-C.

Studies 2 and 3 were conducted in parallel to compare the efficacy of Alum^N^ at higher doses (2,160 μg/dose) and MF59 against a Tier 1 SHIV-C challenge in Chinese rhesus macaques. Because SIV-Alum^Alh12.5^ decreases the risk of SIV_mac251_ acquisition in Chinese macaques, we can exclude the macaques’ origin as a factor in the inability of Alum^N10.8^ to decrease the risk of SHIV-C acquisition. The Alum^N10.8^ and MF59 regimens included an additional immunization with ALVAC-HIV and two additional gp120 protein boosts not performed in Study 1. However, the total amount of protein used over the course of immunization (800 μg) did not differ among these studies because the dose of each gp120 boost was halved (Table 1). Interestingly, tripling the dose of Aluminum hydroxide in Alum^N10.8^ resulted in the elicitation of binding antibodies to V2 linear peptides that strongly correlated with delayed Tier 1 SHIV-C acquisition (R = 0.9, *P* = 0.003). Despite this strong immune correlate of risk in the Alum^N10.8^ study, the overall risk of Tier 1 SHIV-C acquisition did not differ significantly between vaccinated and control animals, likely due to the small number of animals used in this study (n = 8) and the limited efficacy of this vaccine regimen. When contrasted with Study 1, the results of Study 2 suggest that a high alum dose may also be important for the induction of protective IgG to V2 in this animal species.

Several lines of evidence provide support for the importance of the Alum dose. First, we demonstrated that *in vitro* IL-1β induction is reduced following stimulation of PBMCs with a low dose of Alum^N^. Alum is a potent inducer of inflammasome activation and IL-1β production, which in turn triggers innate monocyte memory by causing emergency myelopoiesis, hypoxia, and lasting epigenetic changes [39] in CXCR4^+^ pre-monocytes. Second, our prior studies with the ALVAC-SIV-based vaccine in the SIV_mac251_ model demonstrated a strong monocyte signature, confirmed by systems biology, that correlated with a decreased risk of SIV_mac251_ acquisition [17]. This suggests the hypothesis that the lack of protection in the Alum^N1.9^ and Alum^N10.8^ arms presented here may be due to an insufficient induction of monocyte innate memory, which is itself a key component of the reduced risk of virus acquisition afforded by the ALVAC/gp120/Alum vaccine modalities.

Another contributing factor appears to be the balance of IgG and IgA responses to V2. Results from recent studies have highlighted the importance of humoral responses to the V2 region of the SIV and HIV-1 envelopes that are correlated with protection [7, 18, 36]. In particular, IgG targeting the V2 region of gp120 are associated with protection [18, 21] when using ALVAC-based vaccine modalities. Data from both clinical and preclinical studies suggest antibody-dependent cell-mediated cytotoxicity (ADCC) mediated by non-neutralizing IgG as another correlate of reduced risk of HIV-1 and SIV acquisition [7, 18, 21]. By contrast, gp120-specific plasma IgA directed to C1 were correlated with increased risk of HIV-1 acquisition in the RV144 trial [21], potentially linked to the inhibition of ADCC-mediating antibody binding to cells infected with HIV-1.

The MF59 and Alum adjuvants induced gp120 specific antibodies that differed in their Fc functional profile in Chinese macaques, as previously observed in Indian macaques [18]. As was the case in the SIV_mac251_ studies performed with these vaccine modalities, however, no correlation was found between the inferred Fc functions and the risk of SHIV-C acquisition.

The finding that the MF59-based vaccine strategy tested in Study 3 was highly effective in reducing the risk of Tier 1 SHIV-C even with a low number of animals is encouraging. Yet, the relevance of the Tier 1 SHIV-C model to humans is uncertain since more than 99% of circulating HIV-1 strains in humans are neutralization-resistant. However, MF59 induced an inflammatory profile that correlated with the titers of neutralizing antibodies, suggesting that immunogens able to induce cross-clade neutralizing antibodies other than those used here may be potentiated by this adjuvant. There has been increasing interest in testing HIV-1 neutralizing antibodies that might prevent or control viral infection, but only 10–30% of chronically infected individuals develop cross-neutralizing antibodies for different HIV-1 clades. Recently, a unique inflammatory signature in HIV-1 controllers was shown to be associated with the production of broadly neutralizing antibodies [40].

Expanding our understanding of the immune mechanisms that affect the risk of HIV and SIV acquisition is necessary to properly guide the choice of all components of an effective vaccine. Hopefully, the accumulation of results from human trials will soon identify a suitable and predictive animal model to hasten the improvement of HIV vaccine candidates.

## Methods

### Vaccines and Tier 1 and Tier 2 SHIV-C challenges

In Study 1, 47 juvenile, female Indian rhesus macaques were housed at the NIH and randomized into two arms controlled for MHC-I allele representation, age, and weight. Twenty (n = 20) macaques were administered Alum^N^ and used as controls. Twenty-seven (n = 27) others were immunized at weeks 0, 4, 12, and 24 with intramuscular inoculations of 10^8^ PFU of ALVAC vCP172 [41] and ALVAC vCP2438 [12]. ALVAC vCP172 expresses SIV *gag-pol*. ALVAC-HIV (vCP2438) is considered equivalent to vCP1521 as used in the RV144 vaccine regimen, but the CRF01_AE gp120 insert (92TH023) was exchanged for a subtype C gp120 insert (96ZM651) to match the virus strain circulating in South Africa that was fused to the clade B LAI *env* trans-membrane portion. Additionally, vCP2438 expressed the clade B HXB2 Gag and Protease [12]. At weeks 12 and 24, the treated group was given a bivalent monomeric gp120 protein boost in the thigh opposite of the one that received the ALVAC. The boost formulation contained 200 μg each of clade C TV1 and 1086 in Alum hydroxide manufactured by Novartis through a proprietary process (Alum^N^). Per Novartis’s recommendation, the proteins were prepared over the course of 2 days through the following process. The day before vaccination, the TV1 gp120 and 1086 gp120 proteins were thawed at room temperature (RT). Alum Formulation Buffer (AFB) and Alum^N^ were removed from 4°C and gently inverted 10 times. Next, 3.5 ml of Alum^N^ were added to the AFB vial. The solution was gently inverted 10 times to ensure a uniform mix. Then, 1.591 ml of thawed TV1 gp120 was added to each vial of AFB + Alum, and 1.129 ml of thawed 1086 gp120 were added to each vial of AFB + Alum^N^. The solution was gently inverted 10 times to mix immediately after addition, then stirred gently for 5 min. The vial was tightly capped and transferred to a cold room (4°C) for overnight stirring. The vial was gently shaken on the day of the immunization. The total aluminum contained in each gp120 dose was 0.75 mg, at a ratio of 1.9:1 adjuvant:gp120 (Alum^N1.9^, see Table 1). 1.0 mL (200 μg of each gp120 dose) of this final mixture was administered to each animal intramuscularly in the opposite thigh of the vector immunization within four hours of opening the vial. *In vitro* characterization of the formulation (pH, osmolality, endotoxin and SDS PAGE Gels) was performed by Novartis on the amount left over after vaccination to control for protein integrity. The study included 20 simultaneous controls that each received 1 ml of Alum^N^ (Fig 1A). All controls were housed in the same facility and were challenged by the same operators with the same challenge virus stock. Four weeks after the last immunization, all 27 vaccinated animals and 20 controls were challenged vaginally with the Tier 2 SHIV-1157ipd3N4 grown in rhesus macaque PBMCs (TCID_50_ in TZM-bl cells: 1.56 × 10^5^/ml and TCID_50_ in rhesus PBMC: 1.02 × 10^4^/ml) six times at a 1:100 dilution, then eight times at a dilution 1:50, and for three final challenges at a maximum dilution of 1:5. Animals that tested negative for SHIV RNA in plasma were re-challenged and exposed to the virus for a maximum of 17 weekly challenges.

In Studies 2 and 3, 28 juvenile, female Chinese rhesus macaques were hosted at ABL and randomized into three groups: Study 2 (n = 10), Study 3 (n = 10), and a control group (n = 8). The macaques in Studies 2 and 3 were immunized at weeks 0, 4, 24, 49, and 111 intramuscularly with 10^8^ PFU of the same ALVAC vCP172 and ALVAC vCP2438 used in Study 1. At weeks 12, 24, 49, and 111, the treated groups were separated and given a bivalent monomeric gp120 protein boost in the opposite thigh of the vector immunization. The protein boost administered in Study 2 contained 100 μg each of clade C TV1 and 1086 in Alum^N^ (Fig 1B), whereas Study 3 received 100 μg each of clade C TV1 and 1086 in MF59 (Fig 1C). Both 1086 Alum^N^ and MF59 were manufactured by a proprietary Novartis process. The proteins adjuvanted with Alum^N^ were formulated as in Study 1, but with a higher level of Alum salt. The total aluminum contained in each gp120 dose was 2.16 mg, with 10.8:1 adjuvant:gp120 (Alum^N10.8^, see Table 1). Per Novartis’s recommendation, the proteins adjuvanted with MF59 were formulated through the following process: TV1 gp120, 1086 gp120, and Buffer M were thawed at RT. The buffer M vial contained buffer salts to achieve an isotonic formulation with a final concentration of 10 Mm Phosphate buffer (pH 7.4). Once the three vials were thawed, the empty formulation vial and one vial of MF59C.1 (adjuvant) were removed from refrigeration and mixed by repeated gentle swirling and inversion. Using a suitable sterile syringe, 2.380 ml of buffer M were withdrawn and transferred into the formulation vial, followed by 4.320 mL of MF59C.1. The contents were mixed gently. In similar fashion, 1.382 ml of TV 1 gp120 and 0.558 ml of 1086 gp120 were added into the formulation vial and mixed gently. The admixed vaccine was stored at RT until administration, no longer than 4 h after the addition of MF59. Prior to administration, the vial was shaken gently and, using a sterile syringe, 0.5 ml of the vaccine was drawn and injected into each animal. The syringes were inverted gently three times prior to each administration. All injections were administered intramuscularly.

The control group was added to the study before intravaginal challenges began. All animals were housed in the same facility and challenged by the same operators with the same challenge virus stock. Two animals perished in each group during the immunization phase due to ailments unrelated to the study. The remaining 8 animals in each group were challenged four weeks after the last immunization and 115 weeks after the first ALVAC administration with Tier 1 SHIV-1157ipEL-p. Challenges were administered via the vaginal route 12 times using SHIV-1157ipEL-p with a TCID_50_ in TZM-bl cells of 3.2 × 10^5^/ml at a dilution 1:10 (Fig 1B and 1C). Because MF59 is a protein dose-sparing adjuvant, the amount of gp120 in the boost used in Study 3 was halved compared to the one used in Study 1 (Study 1, 200 μg; Study 3, 100 μg). Animals that tested negative for SHIV RNA in plasma were re-challenged and exposed to the virus for a maximum of 12 weekly challenges. Hormone treatment to synchronize menstrual cycles and thin vaginal epithelium was not used in any group.

### Measurement of viral RNA, and CD4 count

Plasma SHIV RNA was quantified by nucleic acid sequence-based amplification (NASBA), as described in previous studies [42]. The normalized value of the proviral load was calculated as copy number/Mac albumin gene copy number × 2 × 10^6^, and expressed as the number of SHIV DNA copies/ml. CD4^+^ T cell counts were determined from whole blood by flow cytometry, as previously described [43].

### ELISA for TV1 and 1086 in plasma

An enzyme-linked immunosorbent assay (ELISA) was used to detect SHIV gp120 binding antibodies in blood as previously described [44] and to detect binding to overlapping peptides spanning gp120. A serial dilution of plasma was added to microtiter plates coated with the Env protein or individual peptides of natively purified gp120 SHIVs, as determined by the antibody titer. Absorbance at OD450 nm was reported for peptide mapping. For binding antibodies to gp120, the endpoint titers were defined as two times the OD450 nm of the negative control serum.

### Neutralizing antibodies

Neutralization was measured as a reduction in luciferase reporter gene expression after a single round of infection in TZM-bl or Rhesus cells, as described previously [45, 46]. TZM-bl cells were obtained from J. Kappes and X. Wu of the NIH AIDS Research and Reference Reagent Program. Briefly, a pre-titrated dose of Env-pseudotyped virus or replication-competent challenge stock was incubated with serial 3-fold dilutions of test sample in duplicate in a total volume of 150 μl for 1 h at 37°C in 96-well flat-bottom culture plates. Freshly trypsinized cells (10,000 cells in 100 μl of growth medium containing 20 μg/ml DEAE dextran) were added to each well. One set of control wells received cells + virus (virus control) and another set received cells only (background control). After 48 h incubation, the cells were lysed by the addition of Britelite (PerkinElmer Life Sciences, Waltham, Massachusetts, USA), and three quarters of the cell lysate were transferred to a 96-well black solid plate (Corning Costar, Tewksbury, Massachusetts, USA) for luminescence measurement. Neutralization titers are the serum dilution (ID50) at which relative luminescence units (RLU) were reduced by 50% compared to virus control wells after subtraction of background RLUs. The SHIV-1157ipd3N4 and SHIV-1157ipEL-p challenge stocks used in this assay and in the challenges were respectively obtained from N. Miller (NIH Division of AIDS) and the NIH AIDS Reagent Program. Challenge stocks were expanded on rhesus PBMCs and titered before use.

### Particle enhanced immunoturbidimetric assay for CRP

Rhesus macaque CRP agglutinates with latex particles coated with monoclonal anti-human CRP antibodies. CRP values for rhesus plasma samples were detected and measured on a Roche/Hitachi cobas c501 analyzer. The precipitate was determined turbidimetrically. The following two reagents were added: Reagent R1 (82 μl of R1 + 42 μl of H_2_O) containing TRIS buffer with bovine serum albumin and mouse immunoglobulins with preservative and stabilizers; R2 (28 μl of R2 + 20 μl of H_2_O) containing Latex particles coated with anti-CRP (mouse) in glycine buffer with preservative and stabilizers. K_2_-EDTA plasma isolated from whole blood was used in this assay. Samples were loaded with a reaction time of 10 s each and read at a wavelenght of 546 nm. Results were expressed as a concentration of mg/L, nmol/L, or mg/dL. The reader was calibrated for new reagents with multiple solutions to determine the standard concentrations for the 6-point calibration curve. This method has been standardized against the reference preparation of the IRMM (Institute for Reference Materials and Measurements) BCR470/CRM470 (RPPHS–Reference Preparation for Proteins in Human Serum). LIQUICHEK was used as a quality control serum (IMMUNOLOGY CONTROL, Levels 1 [cat. #591], 2 [cat. #592], & 3[cat. #593], BIO RAD Laboratories Diagnostics Group, Irvine, California, USA). Measuring range: 0.15–20.0 mg/L (1.43–190 nmol/L, 0.015–2.0 mg/dL)

### Plasmablast staining in macaque blood

The frequency of plasmablasts (PBs) was measured in the blood of 12 macaques vaccinated with ALVAC-SHIV/gp120 Alum before vaccination and 7 days after the last immunization. Cells were stained with CD3 (Clone SP34-2), CD14 (Clone M5E2), CD16 (Clone 3G8), and CD56 (Clone B159), all conjugated in ALEXAFluor700 (BD Biosciences; San Jose, California, USA), PE-Cy5-CD19 (Clone J3-119, Beckman Coulter; Brea, California, USA), QDOT-650-CD20 (Clone 2H7, eBioscience; San Diego, California, USA), FITC-CD38 (Clone AT-1, StemCell; Massachusetts, USA), BV421-CD39 (Clone MOCP-21, BioLegend; San Diego, California, USA), PE-Ki67 (Clone B56, BD Biosciences), and anti-CXCR3 (CXCR3/CD183 PE-CF594 conjugated). Clone 1C6BD (Biosciences cat. #11718; anti-α4β7) was kindly provided by Dr. Ansari through the NIH AIDS Reagent Program, Division of AIDS, NIAID. Cells were permeabilized with Cytofix/Cytoperm (BD Biosciences). Acquisition was performed on an LSRII cytometer (BD Biosciences) and data were analyzed by FlowJo software (TreeStar; Ashland, Oregon, USA). Plasmablasts were gated as previously described: lineage-(CD3^-^ / CD14^-^ / CD16^-^ / CD56^+^) / (CD19^+^/ CD20^+^ / (CD21^-^CD27^+^) / Ki67^+/++^ / (CD38^+/++^ / CD39^+^) [18]. The frequency of PBs expressing CXCR3 or α_4_β_7_ was then calculated.

### Plasma and mucosal antibody binding and specific activity

Binding and linear epitope mapping of vaccine-induced IgG and IgA in plasma and/or mucosal samples to HIV-1 Envs was measured by binding antibody multiplex assay (BAMA) and peptide microarray. HIV-1 V1/V2-specific BAMA assay was performed as previously described [21] using gp70-V1/V2 scaffolds. Mucosal samples were filtered and concentrated. For vaccine-induced IgA response evaluation, IgG was depleted from plasma or mucosal samples. Total IgG and IgA concentrations in the prepared mucosal samples were determined using a custom total rhesus IgG and IgA ELISA assay. Specific activity in mucosal BAMA was defined as MFI × dilution/total IgG concentration (μg/ml). Linear epitope mapping microarray was performed as previously described [47, 48]. Intensity of binding to each peptide was baseline subtracted (mean of baseline binding + 3 × s.d. of triplicate tests, or at least 200), and then normalized by total IgG concentration per sample to obtain Specific Activity. Specific Activity was calculated as Baseline subtracted intensity × dilution/total IgG concentration (μg/ml).

### Reagents and surface plasmon resonance for cyclic V2

To deactivate the complements and remove lipid contents, sera or mucosal secretions were heated at 56°C for 45 min, followed by centrifugation at 16,000 × g at 4°C for 20 min. The supernatants were collected and stored at -80°C for Biacore assay. The immunogenicity assessment for cyclic V2 peptides was conducted using the Biacore 4000 system [17, 49, 50]. The immobilizations were completed in 10 mM HEPES and 150 mM NaCl (pH 7.4) using a standard amine coupling kit. The CM7-S series chip surface was activated with a 1:1 mixture of 0.4 M 1-ethyl-3-(3-dimethylaminopropyl) carbodiimide hydrochloride (EDC) and 0.1 M N-hydroxysuccinimide (NHS) for 600 s. Following this, 1 μM Streptavidin was immobilized in 10 mM sodium acetate (pH 4.5) for 720 s (11,400–12,300 RU). The immobilized surface was then deactivated by 1.0 M ethanolamine-HCl (pH 8.5) for 600 s. Spot 3 in each flow cell was left unmodified to serve as a reference. After the surface deactivation, 2.5 μM cyclic biotinylated peptide of AE.92TH023.V2 (cbV2_AE; 4,700–4,800 RU), B.MN.V2 (cbV2.MN; 4,000 RU), B.caseA2.V2 (cbV2.BcaseA2; 5,200–5,400 RU), or C.1086.V2 (cbV2.C; 4,900 RU) were captured. Following surface preparation, the heat-inactivated plasma/secretions were diluted (1:40) in 10 mM HEPES and 150 mM NaCl (pH 7.4). The diluted plasma was dispensed onto the captured surface for 300 s, then allowed a 30 s dissociation period. The bound antibodies were then enhanced by the injection of a secondary goat anti-human IgG antibody (30 μg/ml) for 360 s. To regenerate the bound surface, 175 mM HCl was injected for 60 s. Each sample was tested in quadruplicate for each peptide at a rate of 10 Hz at an analysis temperature of 25°C. All sample injections were conducted at a flow rate of 10 μl/min. Data analysis was performed using Biacore 4000 Evaluation Software v. 4.1 with double subtractions for unmodified surfaces and the buffer for blank.

### Cell culture, adjuvant stimulation, and Luminex

PBMCs obtained from human donors and Indian and Chinese rhesus macaques were isolated from Leukopacks by Ficoll-Histopaque density. PBMCs were washed, resuspended in complete medium (RPMI 1640 medium containing 10% FBS and 1% Anti-anti), and used immediately. Cells were prepared for cell culture in 24-well plates at a concentration of 5 × 10^6^ PBMCs/well. Briefly, 5 × 10^6^ PBMCs were suspended in each well at different conditions in a final volume of 500 μl. PBMCs cultured in medium alone were used as negative controls, and PBMCs stimulated with LPS (1.2 ng/ml) were used as positive controls. In stimulations with Alum Alhydrogel or Alum Novartis (Alum^N^), 5 × 10^6^ PBMCs were plated together with the negative and positive controls in 24-well plates in 0.5 ml of RPMI 1640 / 10% FBS / 1% AA and stimulated with the two adjuvants. Either 78 μg of Alum Alhydrogel or Alum^N^ were used for each well. In a separate well, cells were cultured with 7.8 μg of Alum Novartis to maintain the proportion of Alum used in vivo. PBMCs were cultured for 24 h in a humidified atmosphere of 5% CO_2_ at 37°C. Conditions were chosen based on previously published data [51]. PBMCs were harvested and centrifuged at 2000 rpm for 6 min at 4°C. Supernatant was collected, aliquoted, and frozen at -80°C for Luminex analysis. Cryopreserved supernatants were analyzed using three MILLIPLEX Non-Human Primate Multiplex assays (EMD Millipore Corporation, Billerica, MD). The following targets were assayed following manufacturer instructions: IL-1β, IL-2, IL-4, IL-6, IL- 8, IL-10, IL-13, IL-17A, IFN-γ, MCP-1, MIP-1a (cat. #PRCYTOMAG-40K-11). After thawing the samples on ice, 25 μl of each supernatant was loaded into the well and briefly mixed with 25 μl of assay buffer and 25 μl of magnetic beads. The plates were incubated under agitation at 4°C for 18 hours. After washing, 25 μl of detection antibody was added to each well and incubated for 1 h RT. Next, 25 μl of Streptavidin-PE was added to each well and incubated for 30 m RT. Finally, wells were washed and resuspended in 150 μl of blank fluid according to the manufacturer’s instructions. Samples were acquired on a Bio-Plex 200 System (Bio-Rad; Hercules, California, USA).

### Ethics statement

All animals used in this study were colony-bred female rhesus macaques (*Macaca mulatta*) obtained from Covance Research Products (Alice, Texas, USA). Macaques used in Study 1 were of Indian origin and animals used in Studies 2 and 3 were of Chinese origin.

Animals in Study 1 were housed at the National Institutes of Health (Protocol VB008) and those of Studies 2 and 3 were housed at the BIOQUAL nonhuman primate facility (Protocol P169). Animals were cared for in accordance with American Association for the Accreditation of Laboratory Animal Care (AAALAC) standards in an AAALAC-accredited facility (Animal Welfare Assurance A4149-01). All animal care and procedures were carried out under protocols approved by the NCI or NIAID Animal Care and Use Committees (ACUC; Protocol numbers: P169 and VB008). Animals were closely monitored daily for any signs of illness, and appropriate medical care was provided as needed. Animals were housed individually during the challenge phase to reduce the risk of transmission of SIV or other viruses. All clinical procedures, including biopsy collection, administration of anesthesia and analgesics, and euthanasia, were carried out under the direction of a laboratory animal veterinarian. Steps were taken to ensure the welfare of the animals and minimize discomfort of all animals used in this study. Animals were fed daily with a fresh diet of primate biscuits, fruit, peanuts, and other food items to maintain body weight or normal growth. Animals were monitored for mental health and provided with physical enrichment including sanitized toys, destructible environments (cardboard and other paper products), and audio stimulation. Leukopacks from humans were obtained from healthy donors from the NIH Blood Bank.

## Supporting information

S1 Fig Specific IgG activity to overlapping peptides that encompass the 1086 V1/V2 region in Alum^N1.9^. (A) Amino acid sequence (single letter aa code) of 18 overlapping peptides(15-mers) encompassing the V2 of 1086 (V2, sequence underlined). (B, D) V2 specific IgG in plasma (13 animals) paired with those in vaginal secretions (C and E) in 13 of the 27 immunized animals at week 26. Plasma and vaginal IgG-Specific activity was measured as MFI × dilution.total IgG (μg/ml).
(TIF)

S2 Fig V2-specific IgG in the remaining 14 vaccinated animals in Alum^N1.9^. V2 specific IgG in plasma of the remaining 14 of the 27 immunized animals for which data on vaginal secretion were not available (week 26). Plasma IgG-Specific activity was measured as MFI × dilution.total IgG (μg/ml). Arrows indicate low, but specific IgG activity.
(TIF)

S3 Fig Specific IgG activity to overlapping peptides encompassing the V2 region of 1086 in Alum^N10.8^ and MF59. (A) plasma and vaginal IgG in 9 out of 10 immunized animals in Alum^N10.8^ at week 50. (B) Specific plasma and vaginal IgG in 9 out of 10 immunized animals in MF59 at week 50. All plasma and vaginal IgG-specific activity was measured as MFI × dilution.total IgG (μg/ml). Arrows indicate low, but specific IgG activity.
(TIF)

S4 Fig Inflammation markers and cytokine production in human and macaque cells. (A) Plasma levels of C-Reactive Protein (mg/dl) measured in 9 out of 10 vaccinated animals in both Alum^N10.8^ and MF59 groups at week 50. (B) Plasma levels of CXCL10 (pg/ml) measured in 9 out of 10 vaccinated animals in Alum^N10.8^ and MF59 at week 50. (C) IL-1β, (D) IFN-γ, (E) IL-10, (F) IL-13, and (G) CCL3 production measured in human (empty hexagons), Indian rhesus macaque (dark-top hexagons), and Chinese rhesus macaque (dark-bottom hexagons) PBMCs following *in vitro* stimulation with Alum^Alh^, MF59, and LPS. (H) IL-10, (I) IFN-γ, (J) IL-13, and (K) CCL3 levels measured in human (empty circles), Indian rhesus macaque (dark-top hexagons), and Chinese rhesus macaque (dark-bottom hexagons) PBMCs following stimulation with Alum^Alh^ (78 μg) and Alum^N^ at the same concentration (78 μg) or at 7.8 μg. Horizontal lines represent the median values in all panels.
(TIF)

S5 Fig Human and macaque PBMC cytokine level distribution and production after stimulation with different adjuvants. The violin plots show the distribution of the levels of (A) IL-1β, (B) IFN-γ, (C) IL-10, (D) IL-13, and (E) CCL3 in human (dark grey) and rhesus macaque (light grey) PBMCs. Different stimuli used in culture with PBMCs to induce cytokine productions are shown on the x axis. The thick bar in each plot represents the interquartile range, and the thin line extending from it represents the 95% confidence intervals. The median value is denoted by a dot.
(TIF)

S6 Fig Specific anti-TV1 antibody glycoforms in sera of Alum^N1.9^, Alum^N10.8^, and MF59 studies. Unless stated otherwise, data associated with the Alum^N1.9^ study are represented by full red circles, data from the Alum^N10.8^ study are represented by full green circles, data from the MF59 study are represented by full blue circles, and only available samples (i.e., those not depleted in other experiments) are shown. The number of samples represented in each graph is stated in the figure legend. Serum was tested for the following anti-TV1 antibody glycoforms in vaccinated animals (Alum^N1.9^, n = 27; Alum^N10.8^; n = 9; MF59, n = 9): (A) G0F, (B) G1F, (C) G2F, (D) G2S1F/G0, (E) G2S2F_B, and (F) G2_FB. Statistical analysis was performed using the Mann-Whitney test and corrected by multicomparison analysis via Tukey's range test. Different sugar group patterns mediated or inhibited different antibody functions as described by Ackerman et al. [52].
(TIF)
