# Correction: ALVAC-HIV B/C candidate HIV vaccine efficacy dependent on neutralization profile of challenge virus and adjuvant dose and type

**DOI:** 10.1371/journal.ppat.1008531

**Published:** 2020-04-28

**Authors:** Luca Schifanella, Susan W. Barnett, Massimiliano Bissa, Veronica Galli, Melvin N. Doster, Monica Vaccari, Georgia D. Tomaras, Xiaoying Shen, Sanjay Phogat, Ranajit Pal, David C. Montefiori, Celia C. LaBranche, Mangala Rao, Hung V. Trinh, Robyn Washington-Parks, Namal P. M. Liyanage, Giacomo Gorini, Dallas R. Brown, Frank Liang, Karin Loré, David J. Venzon, William Magnanelli, Michelle Metrinko, Josh Kramer, Matthew Breed, Galit Alter, Ruth M. Ruprecht, Genoveffa Franchini

Dr. Giacomo Gorini is not included in the author byline. He should be listed as the seventeenth author and affiliated with Animal Models and Retroviral Vaccines Section, Vaccine Branch, Center for Cancer Research, National Cancer Institute, Bethesda, Maryland, United States of America. The contribution of this author is as follows: Methodology.
