# Supplementary figures and images for: ALVAC-HIV B/C candidate HIV vaccine efficacy dependent on neutralization profile of challenge virus and adjuvant dose and type
Source: PLoS Pathog. 2019 Dec 3;15(12):e1008121. doi: 10.1371/journal.ppat.1008121 (PMC6890176; doi:10.1371/journal.ppat.1008121)

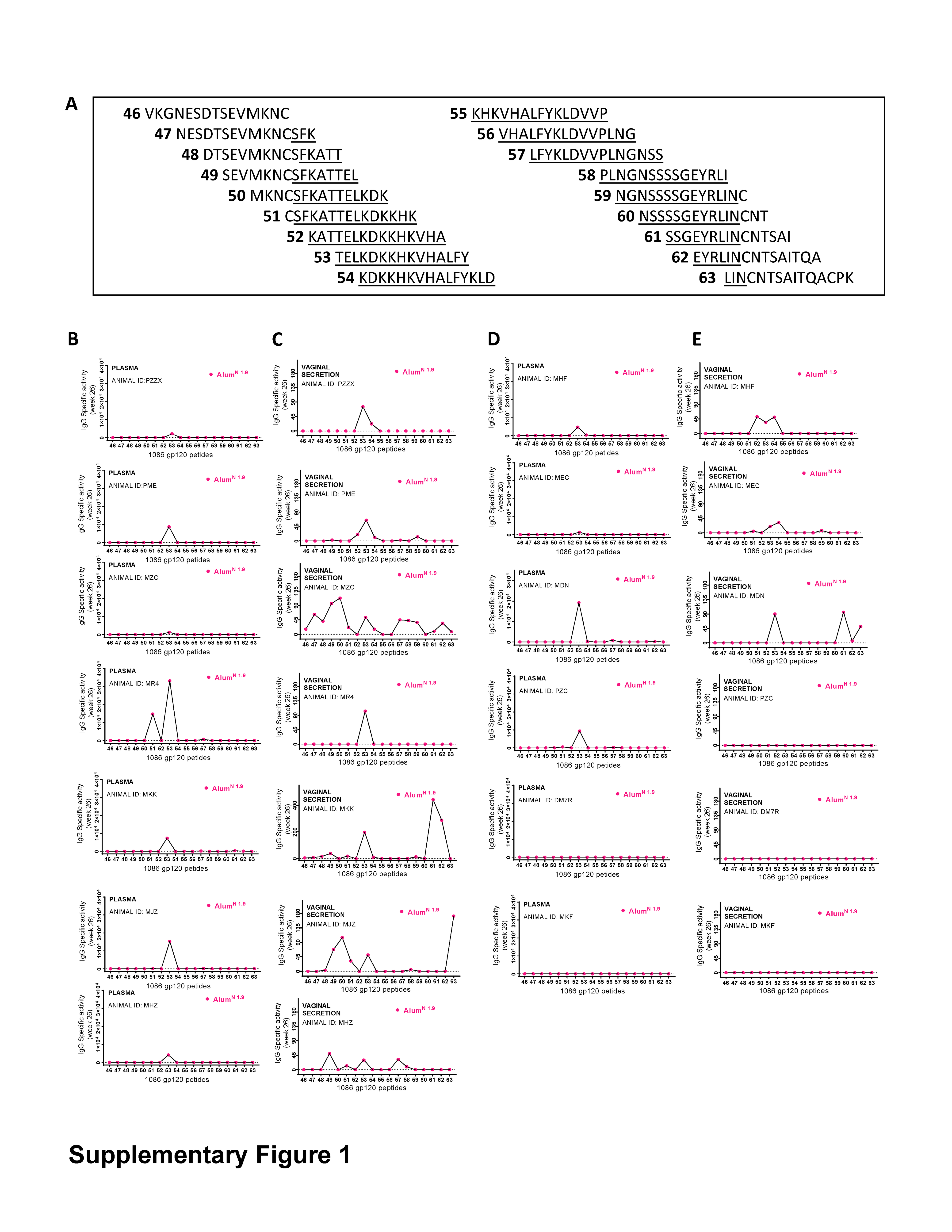

Supplement: S1 Fig — (A) Amino acid sequence (single letter aa code) of 18 overlapping peptides(15-mers) encompassing the V2 of 1086 (V2, sequence underlined). (B, D) V2 specific IgG in plasma (13 animals) paired with those in vaginal secretions (C and E) in 13 of the 27 immunized animals at week 26. Plasma and vaginal IgG-Specific activity was measured as MFI × dilution.total IgG (μg/ml). (TIF) [file ppat.1008121.s001.tif]

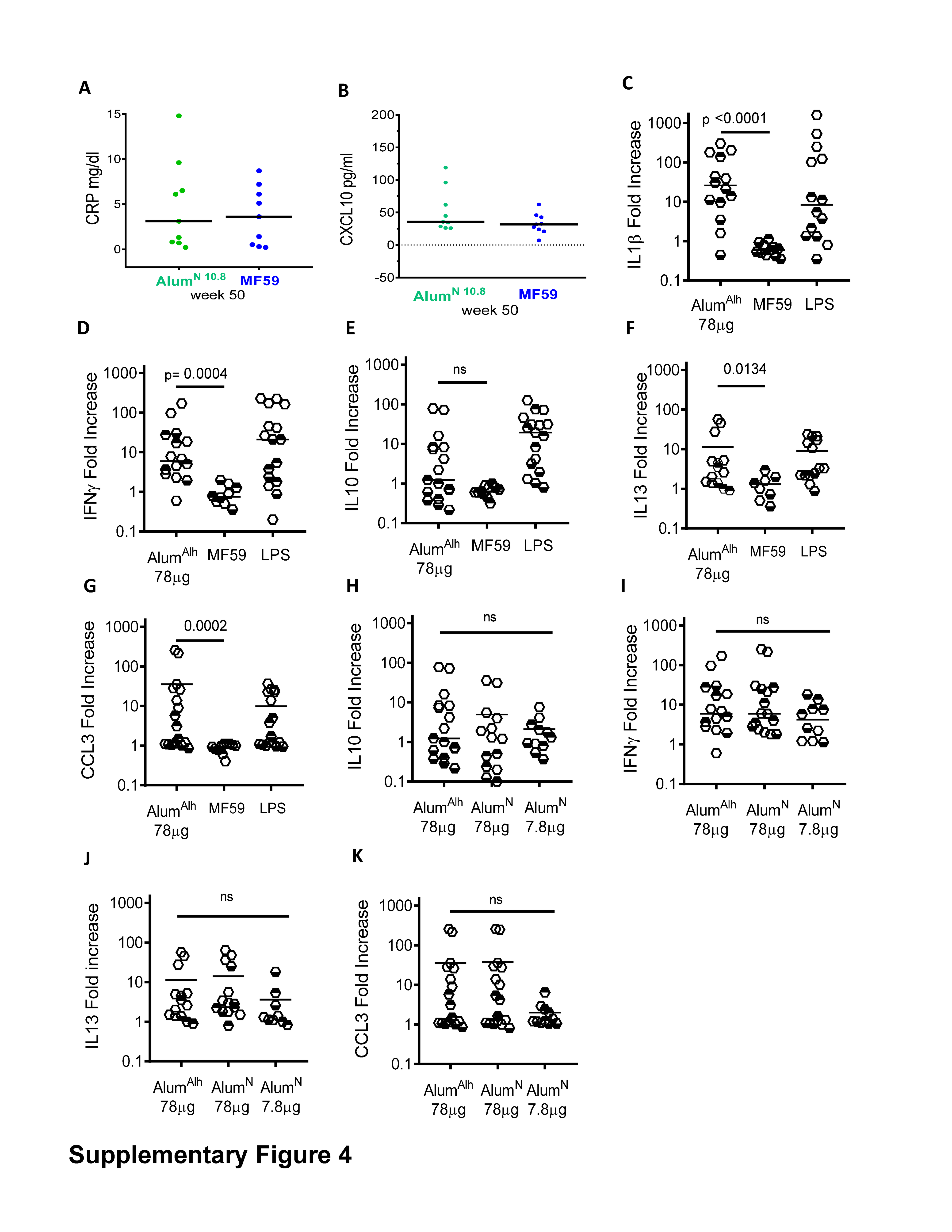

Supplement: S2 Fig — V2 specific IgG in plasma of the remaining 14 of the 27 immunized animals for which data on vaginal secretion were not available (week 26). Plasma IgG-Specific activity was measured as MFI × dilution.total IgG (μg/ml). Arrows indicate low, but specific IgG activity. (TIF) [file ppat.1008121.s002.tif]

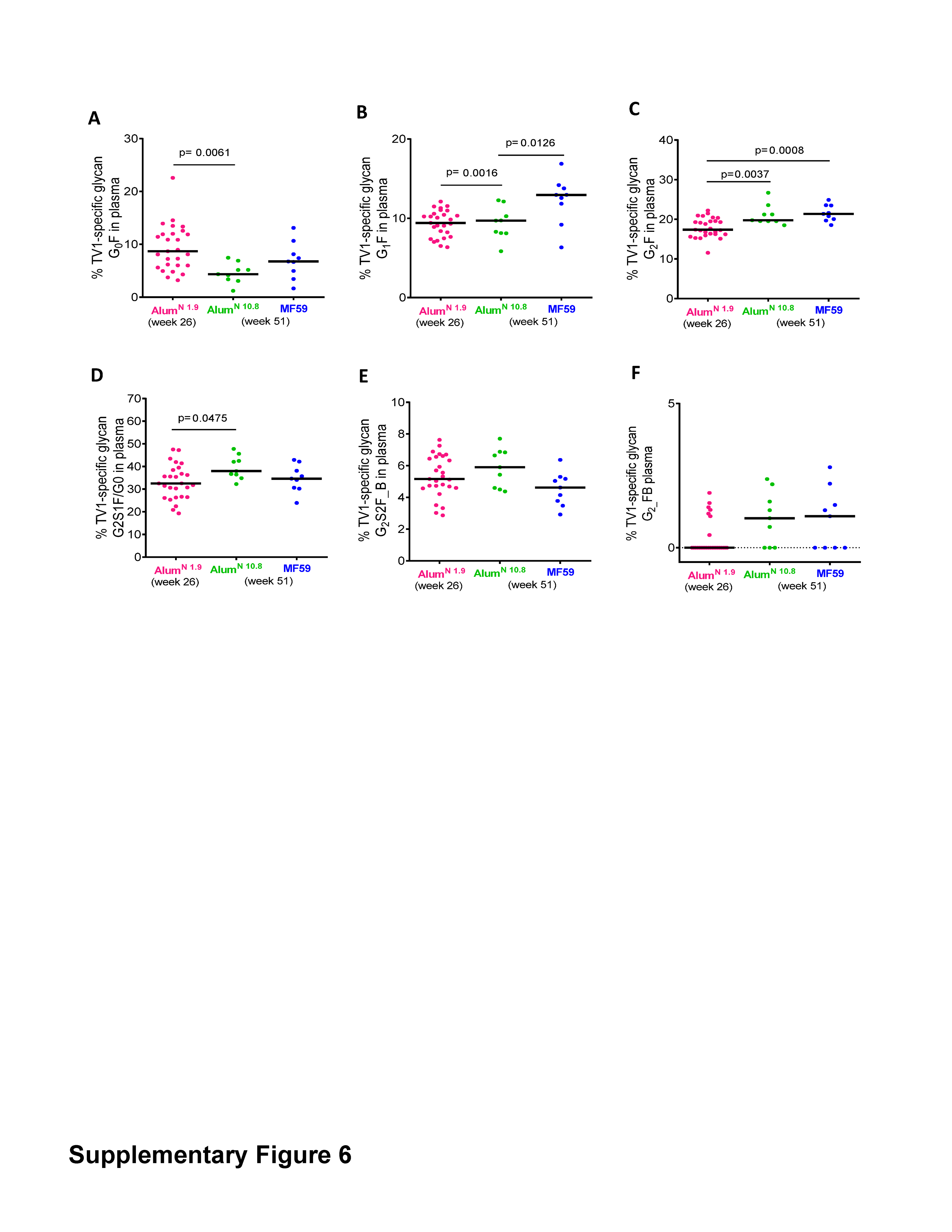

Supplement: S3 Fig — (A) plasma and vaginal IgG in 9 out of 10 immunized animals in AlumN10.8 at week 50. (B) Specific plasma and vaginal IgG in 9 out of 10 immunized animals in MF59 at week 50. All plasma and vaginal IgG-specific activity was measured as MFI × dilution.total IgG (μg/ml). Arrows indicate low, but specific IgG activity. (TIF) [file ppat.1008121.s003.tif]

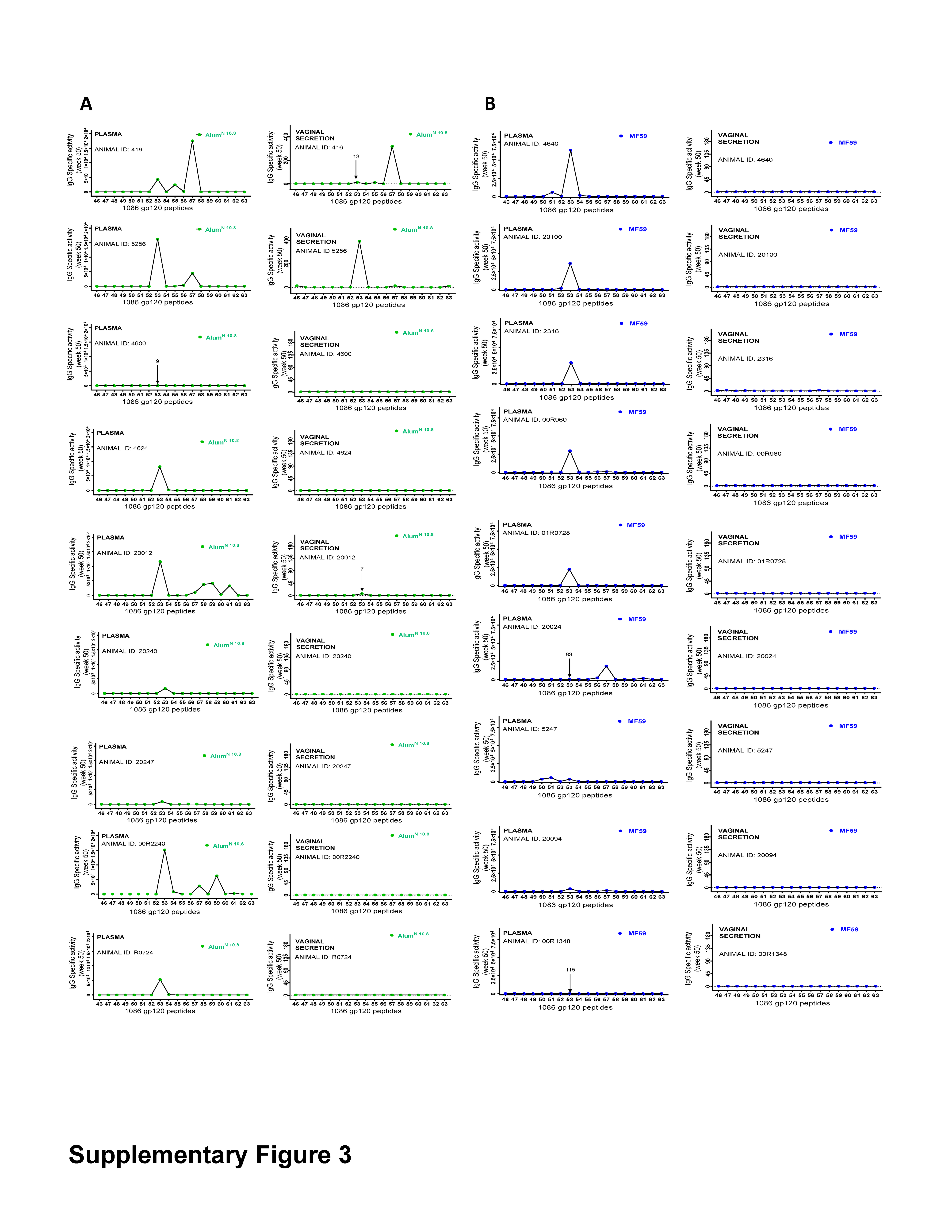

Supplement: S4 Fig — (A) Plasma levels of C-Reactive Protein (mg/dl) measured in 9 out of 10 vaccinated animals in both AlumN10.8 and MF59 groups at week 50. (B) Plasma levels of CXCL10 (pg/ml) measured in 9 out of 10 vaccinated animals in AlumN10.8 and MF59 at week 50. (C) IL-1β, (D) IFN-γ, (E) IL-10, (F) IL-13, and (G) CCL3 production measured in human (empty hexagons), Indian rhesus macaque (dark-top hexagons), and Chinese rhesus macaque (dark-bottom hexagons) PBMCs following in vitro stimulation with AlumAlh, MF59, and LPS. (H) IL-10, (I) IFN-γ, (J) IL-13, and (K) CCL3 levels measured in human (empty circles), Indian rhesus macaque (dark-top hexagons), and Chinese rhesus macaque (dark-bottom hexagons) PBMCs following stimulation with AlumAlh (78 μg) and AlumN at the same concentration (78 μg) or at 7.8 μg. Horizontal lines represent the median values in all panels. (TIF) [file ppat.1008121.s004.tif]

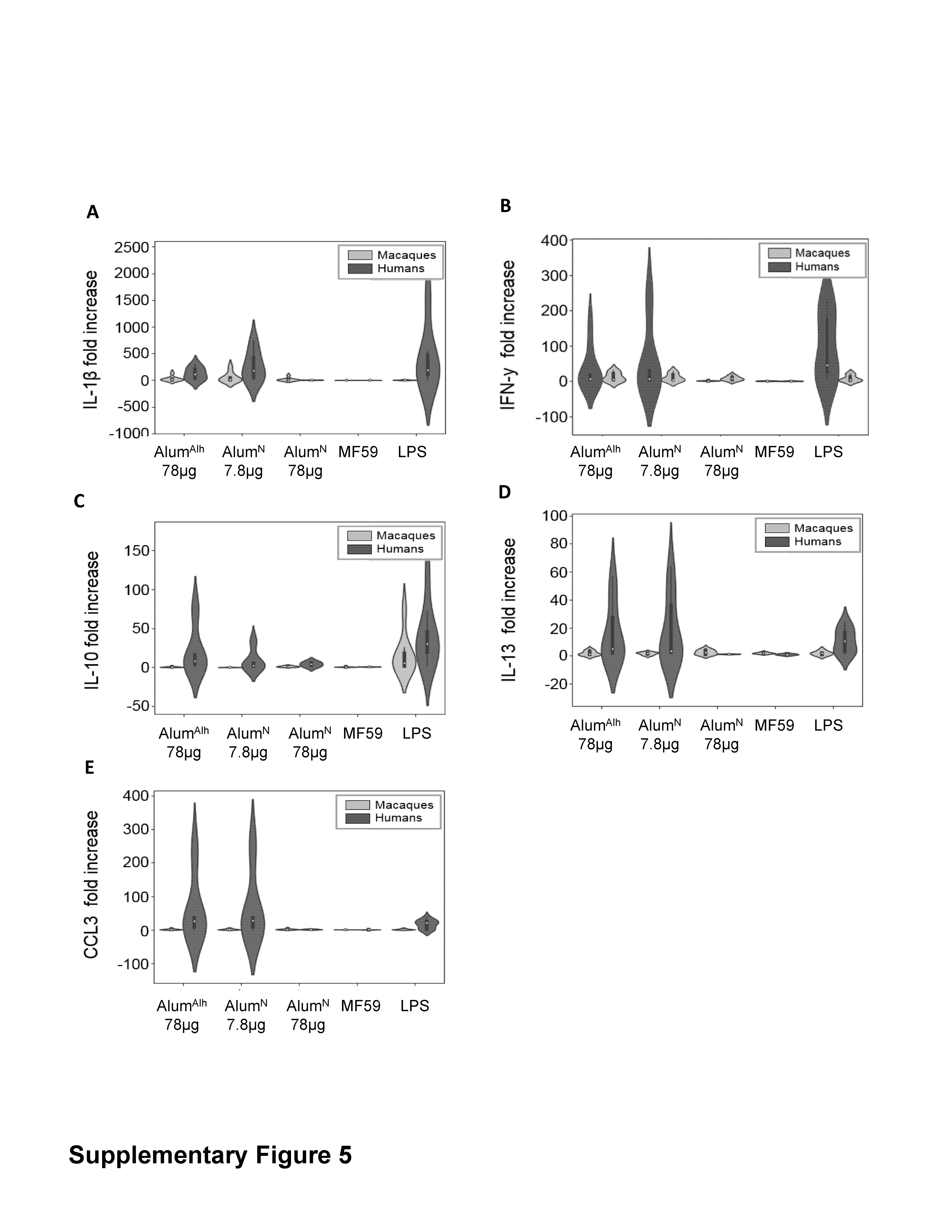

Supplement: S5 Fig — The violin plots show the distribution of the levels of (A) IL-1β, (B) IFN-γ, (C) IL-10, (D) IL-13, and (E) CCL3 in human (dark grey) and rhesus macaque (light grey) PBMCs. Different stimuli used in culture with PBMCs to induce cytokine productions are shown on the x axis. The thick bar in each plot represents the interquartile range, and the thin line extending from it represents the 95% confidence intervals. The median value is denoted by a dot. (TIF) [file ppat.1008121.s005.tif]
